# Regulatory B cell repertoire defects predispose lung cancer patients to immune-related toxicity following checkpoint blockade

**DOI:** 10.1038/s41467-022-30863-x

**Published:** 2022-06-07

**Authors:** Akshay J. Patel, Zena N. Willsmore, Naeem Khan, Alex Richter, Babu Naidu, Mark T. Drayson, Sophie Papa, Andrew Cope, Sophia N. Karagiannis, Esperanza Perucha, Gary W. Middleton

**Affiliations:** 1 Institute of Immunology and Immunotherapy (III), College of Medical and Dental Sciences, University of Birmingham, Birmingham, B15 2TT UK; 2 St. John’s Institute of Dermatology, School of Basic & Medical Biosciences, King’s College London, Guy’s Hospital, King’s College London, London, SE1 9RT UK; 3 Institute of Inflammation and Ageing (IIA), College of Medical Sciences, University of Birmingham, Birmingham, B15 2TT UK; 4 Immunoengineering Group, King’s College London, London, SE1 9RT UK; 5 Department of Medical Oncology, Guy’s and St Thomas’ NHS Trust, London, SE1 9RT UK; 6 Centre for Inflammation Biology and Cancer Immunology, School of Immunology and Microbial Sciences, King’s College London, London, SE1 1UL UK; 7 Centre for Rheumatic Diseases, King’s College London, SE1 1UL London, UK; 8 Breast Cancer Now Research Unit, School of Cancer & Pharmaceutical Sciences, King’s College London, Guy’s Cancer Centre, London, SE1 9RT UK

**Keywords:** Cancer immunotherapy, Non-small-cell lung cancer, Immunosurveillance, Humoral immunity

## Abstract

Checkpoint blockade with Pembrolizumab, has demonstrated durable clinical responses in advanced non-small cell lung cancer, however, treatment is offset by the development of high-grade immune related adverse events (irAEs) in some patients. Here, we show that in these patients a deficient Breg checkpoint fails to limit self-reactive T cell enhanced activity and auto-antibody formation enabled by PD-1/PD-L1 blockade, leading to severe auto-inflammatory sequelae. Principally a failure of IL-10 producing regulatory B cells as demonstrated through functional ex vivo assays and deep phenotyping mass cytometric analysis, is a major and significant finding in patients who develop high-grade irAEs when undergoing treatment with anti-PD1/PD-L1 checkpoint blockade. There is currently a lack of biomarkers to identify a priori those patients at greatest risk of developing severe auto-inflammatory syndrome. Pre-therapy B cell profiling could provide an important tool to identify lung cancer patients at high risk of developing severe irAEs on checkpoint blockade.

## Abstract (web summary)

Immune checkpoint blockade is a promising approach to treat lung cancer, however, immune related adverse events hold back success in some patients. Here authors show that regulatory B cells fail to limit self-reactive T cells in these patients, and B cell phenotyping prior treatment may identify those at risk for these unfavourable outcomes.

## Introduction

Immune checkpoint inhibition has transformed the management of many common cancers. The clinical use of these agents is complicated by the development of a spectrum of immune-related adverse events (irAE). In a meta-analysis of 7936 patients in 48 clinical trials^1^, it was noted that irAE affected multiple organs, including skin, colon, lungs, kidney and endocrine organs. The incidence of these was high ranging from 4.07% for certain endocrinopathies to 50.56% for skin manifestations. These autoimmune sequelae were noted with multiple regimens of checkpoint blockade therapy and were more common and more severe with combination therapy. The most common cause of treatment-related death with anti-PD-1/PD-L1 is pneumonitis (35%), whereas with CTLA-4 blockade 70% of all fatal irAEs are caused by colitis. High-grade toxicities^2,3^ come with the recommendation of discontinuing all further immunotherapy^4^. Given the very widespread use of these agents, the management of severe irAEs has become a significant health care challenge. Predictors of those at particular risk of high-grade toxicity would be of great value in adequately assessing the risk: benefit equation for immunotherapy, to develop vigilant surveillance policies in high-risk patients and potentially to develop preventive strategies.

A large body of evidence has investigated the role of T cells in mediating the anti-tumour response in the context of checkpoint, but it is increasingly clear that B cells are also crucial cellular components contributing to the efficacy of checkpoint blockade^2,3,5^. Higher expression of B-cell-related genes was seen in responders compared to non-responders, as was the density of microenvironmental B cells and tertiary lymphoid structures (TLSs) in the tumour tissue of melanoma patients^2^. Enrichment of the B-cell-rich TLS signature predicted improved survival with immunotherapy in melanoma^3^. Sarcoma immune class (SIC) E is characterised by high expression of a B-cell lineage signature and these patients had the highest response rate (RR) and progression-free survival (PFS) when treated with pembrolizumab^5^.

The role if any of B cells in modulating or mediating severe irAEs is currently underexplored. Bregs are important modulators of the immune response, preventing excessive inflammation and maintaining immune homoeostasis after tissue injury or infection^6,7^. Defects in Breg number and function have been identified in numerous immune-related pathologies such as autoimmune diseases, chronic infection, cancer and transplant rejection^6,8^. Deficiencies in Breg number and function have been implicated in exacerbated intestinal inflammation in models of Crohn’s disease and flare-ups of SLE^9,10^.

The hallmark of Breg identification and enumeration is IL-10 production which is considered the major effector mechanism of their immune suppression. The existence of immune-suppressive B cells was first suggested by the development of an exacerbated form of experimental autoimmune encephalomyelitis (EAE) in B-cell-deficient mice^11^, with subsequent work ascribing a protective role for IL-10-producing B cells in EAE^12^. Various regulatory B-cell subsets have since been described^13,14^ despite the lack of an unequivocal lineage marker by which to capture this heterogeneous population^6^. Unique Breg subsets have been implicated in maintaining immune tolerance in allergen-induced hypersensitivities^15^ and in protecting against allograft rejection in murine models of transplantation, using IL-10 as a biomarker for predicting clinical outcomes in patients undergoing transplantation^16^. More recently, reduced numbers of IL-10-producing B cells have been suggested as contributory to the severity of SARS-CoV-2 infection^17,18^. However, there are also key B-regulatory cell subsets which suppress immune responses independently of IL-10, such as the PDL-1 + B cells^19^. Given their crucial role in suppressing autoimmunity we hypothesise that there might be a defect in Bregs in toxicity-susceptible patients.

In this study, utilising functional ex vivo assays and deep immunophenotyping techniques we explore the role of Bregs in the development of severe immune-related adverse events (irAE) in lung cancer patients treated with anti-PD-1/PD-L1. We show functional and quantitative defects in the B-regulatory cell repertoire at baseline. These data add significantly to our understanding of the aetiopathogenesis of severe toxicity following checkpoint blockade.

## Results

### Study cohort

Our primary study cohort consisted of 46 patients with advanced-stage non-small-cell lung cancer (NSCLC) receiving checkpoint blockade immunotherapy (International Association for the Staging of Lung Cancer (IASLC) stage IIIb/IV) and eight healthy age-matched donors (Supplementary Table 1). Median follow-up was 399 days. All patients who did not develop high-grade toxicity had completed at least 12 cycles of treatment by the time of analysis and with time on treatment across the entire cohort ranging from 331 to 590 days. Eight patients (17.4%) experienced severe grade 3 or greater irAE as defined in the ESMO Clinical Practice Guidelines (Supplementary Table 1)^4^. All irAE were diagnosed using clinico-radiological correlates and required biopsy verification in the case of colitis. These 8 patents constitute the toxicity cohort: the median time to severe toxicity was 42.5 days (range: 31 days (polymyalgia rheumatica)—115 days (colitis)). All patients in the severe toxicity (tox) group discontinued immunotherapy and, in six cases, required the commencement of steroid therapy. The remaining no toxicity (NT) patient group did not experience grade 3/4 toxicity, however, 4 patients in the NT group developed a self-limiting grade 1 skin rash after the first cycle of therapy which resolved with emollients and topical steroids and did not require discontinuation of immunotherapy. There were no significant differences in baseline demographics between the toxicity cohort (*n* = 8) and the NT cohort (*n* = 38) (Supplementary Table 1). At the time of peripheral blood sampling, all patients were treatment naïve (baseline samples, day 0). Post-treatment samples were taken after the first cycle of checkpoint blockade on day 21. There was no significant history of previous cancers, autoimmune phenomena, allergic conditions, current steroid use or recent (within the previous 6 months) vaccination history in our cohort. Clinical responses were determined by Response Evaluation Criteria in Solid Tumours (RECIST) 1.1^20^. For the purposes of data analysis, patients were further categorised as responders (*n* = 26) based on progression-free survival for at least 180 days, while non-responders were patients who had disease progression within 180 days (*n* = 20).

### Ex vivo induction of primary B-cell-derived IL-10 is reduced in patients who develop high-grade irAE on Checkpoint Blockade

**Fig. 1:**
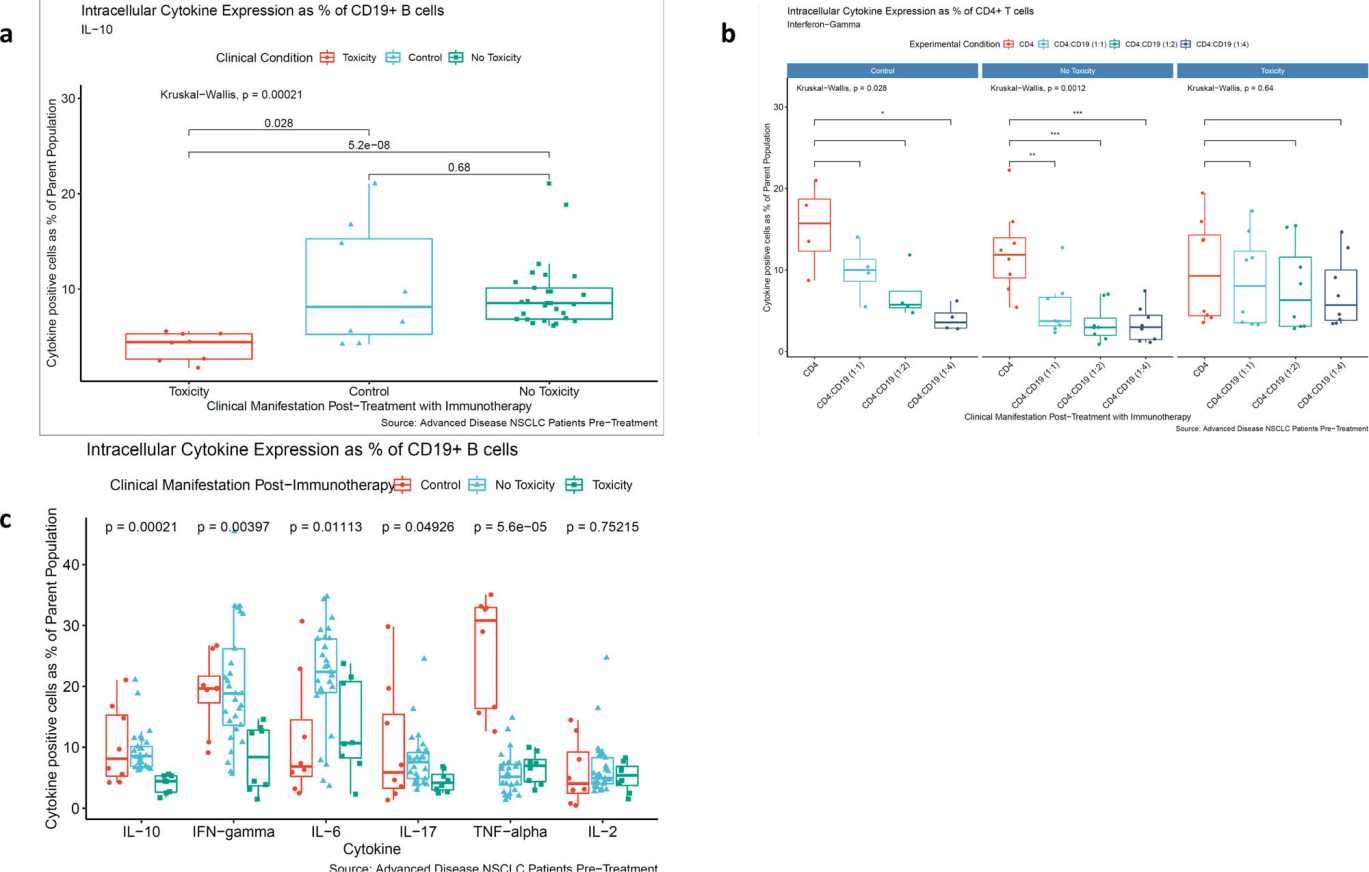
Cytokine induction in primary human B cells from NSCLC patients undergoing checkpoint blockade therapy. **a** CD19 + B-cell IL-10 expression in NSCLC patients stratified by the development of high-grade post-treatment irAE. Data are presented as % of the total CD19 + population, with points indicating individual patients (*n* = 45 biologically independent samples including controls). Box plots are defined as median (centre), with the bounds of the box representing the interquartile range (upper and lower bounds) and the whiskers representing upper and lower extremes. Statistical analysis was conducted using Wilcoxon rank-sums test (to test for specific inter-group differences) and Kruskal–Wallis (to generally test for overall differences between all three groups). All analyses were conducted using a Benjamini–Hochberg multiple comparisons correction, comparing all conditions as indicated. A significance level of <0.05 was adopted. **b** IFNγ production by maximally stimulated CD4 + cells alone and in co-culture with increasing doses of paired autologous primary CD19 + B cells. Data points indicate individual patients (*n* = 20 biologically independent samples including controls). Box plots are defined as median (centre), with the bounds of the box representing the interquartile range (upper and lower bounds) and the whiskers representing upper and lower extremes. Statistical analysis was conducted using Wilcoxon rank-sums test and Kruskal–Wallis followed by a Benjamini–Hochberg multiple comparisons correction, comparing all conditions as indicated; *P < 0.05, **P < 0.01, ***P < 0.001. **c** CD19 + B-cell pan-cytokine expression in NSCLC patients stratified by the development of high-grade post-treatment irAE. Data are presented as % of the total CD19 + population, with points indicating individual patients (*n* = 45 biologically independent samples including controls). Box plots are defined as median (centre), with the bounds of the box representing the interquartile range (upper and lower bounds) and the whiskers representing upper and lower extremes. Statistical analysis was conducted using Kruskal–Wallis (to generally test for overall differences between all three groups). All analyses were conducted using a Benjamini–Hochberg multiple comparisons correction, comparing all conditions as indicated. A significance level of <0.05 was adopted. Source data are provided as a Source Data file.

Functional Breg populations are identified by their ability to produce IL-10 ex vivo upon TLR-9 stimulation^21^. We analysed pre-treatment peripheral blood mononuclear cells (PBMCs) from all the 8 toxicity patients and from 29 NT patients (sample availability prevented analysis of the remaining 9 NT patients) in addition to 8 age-matched healthy controls for comparison. Investigators were blinded to the outcome of the patients by sample pseudonymisation. B-cell effector function (defined as the production of IL-10) was correlated with the development of high-grade irAE in these NSCLC patients. In the NT patients and healthy donors, stimulation of B cells (the CD19 + population in PBMCs) in vitro with phorbol 12-myristate 13-acetate (PMA) and ionomycin after initial stimulation with TLR-9 ligation and IL-2 co-culture resulted in appropriate potent induction of IL-10 at 40 h as measured using intracellular cytokine staining (Fig. 1a and Supplementary Fig. 1). However, the IL-10 response was significantly decreased in those patients who subsequently went on to develop high-grade treatment-related irAE. Baseline IL-10 was measured in these patients from cell culture supernatants (after B-cell induction with CpG and IL-2 only, and prior to PMA/Ionomycin stimulation), and this was significantly lower in toxicity patients (Supplementary Fig. 2).

Bregs can suppress both innate and adaptive cells, including pro-inflammatory cytokine release by monocytes, and a number of T helper (Th) cytokines via IL-10 secretion^6^. We next sought to determine the effect of the Breg IL-10 response on autologous CD4 + T-cell function. TLR-9-activated B cells from patients who did not develop high-grade irAE on treatment with checkpoint blockade therapy were able to suppress Interferon-gamma (IFNγ) production by CD4 + T cells when activated with αCD3 and αCD28 in a dose-dependent manner. A similar result was also observed in B cells from healthy controls (Fig. 1b and Supplementary Fig. 3). However, this dose-dependent suppression was not observed with B cells from patients who subsequently developed high-grade irAEs at either low (1:1) or high (1:4) B-cell to T-cell ratios.

These data demonstrate an inherent functional defect in the B-cell-mediated IL-10 response in lung cancer patients who subsequently experience high-grade autoimmune toxicity on checkpoint blockade therapy, linked to the inability to suppress autologous CD4 + Th1 responses. Given these observations with IL-10, we proceeded to explore a wider array of B-cell-derived cytokines to ask whether this IL-10 production defect was part of a more wider B-cell suppressive programme.

### Pan-cytokine deficit in primary B cells derived from patients who develop high-grade irAE on checkpoint blockade

Using the same patient cohort and methods described above, we examined the following cytokines: IFN-γ, TNF-α, IL-6, IL-17, and IL-2 (Fig. 1c: IL-10 is also included for ease of reference). The pattern for IFN-γ expression was similar to that of IL-10—significantly higher expression in non-toxicity patients when compared with toxicity patients and no difference between healthy donors and no toxicity patients. There was also statistically significantly increased expression of IL-6 and IL-17 in the non-toxicity cohort compared with the toxicity cohort albeit the differences were less marked when compared to IL-10. Healthy controls produced the most TNF-α with no significant difference between toxicity and non-toxicity patients. Thus, there appears to be a polyfunctional failure in B cells derived from toxicity patient that affects both pro- and anti-inflammatory cytokines. Although this seems to be most marked for IL-10, the global cytokine deficit suggests a form of B-cell exhaustion in these patients.

In light of our functional data, we analysed the circulating B-cell repertoire with a focus on suppressive phenotypes in our cohort using high-dimensional deep phenotyping.

### In-depth immunophenotyping of NSCLC patient blood samples using mass cytometry

We performed a large-scale mass cytometry analysis of pre-treatment peripheral blood PBMCs from the entire NSCLC cohort (*n* = 46) and from five healthy age-matched donor samples (Supplementary Table 1). Cells were stained with two bespoke antibody panels devised specifically to address the B-cell phenotype (Supplementary Table 2). The first panel was designed to characterise B cells at various stages of maturation (activated, transitional, marginal zone, follicular, germinal centre (GC), class-switched and plasma) as well as rarer B-cell populations such Bregs. All patient samples were assessed on this first pan-B-cell panel. A second panel was designed to further interrogate Breg phenotypes on a subset of randomly selected patients (*n* = 8). Both panels also included markers for natural killer cells, T cells and granulocytes.

We created two-dimensional maps of the data using dimensionality reduction algorithms (UMAP and diffusion maps). UMAP was used in preference to t-SNE owing to faster run times, especially with large perplexity hyper-parameters and better preservation of the global cellular architecture with competitive visual quality^22^. The arrangement of cells along their development path, with bifurcations along points of differentiation, was displayed using diffusion maps. Distinct cellular phenotypes were determined following the application of the FlowSOM clustering algorithm which employs a Self-Organising Map to determine how markers are behaving on cells in high-dimensional space thus creating well partitioned cellular subsets^23^.

**Fig. 2:**
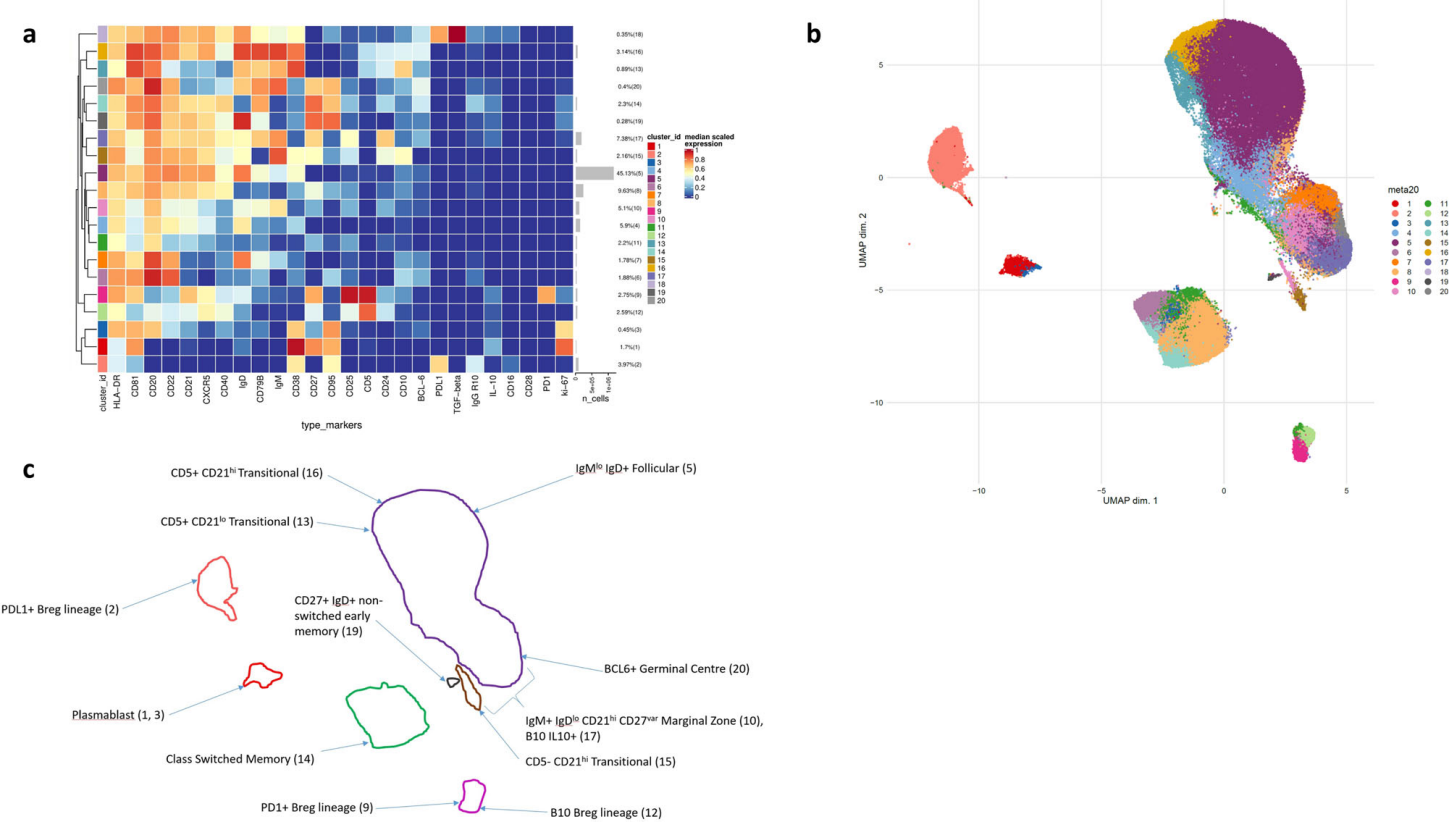
Base B-cell panel phenotyping in NSCLC cohort (*n* = 46). **a** Heatmap showing normalised expression of the base B-cell panel markers for the 20 B-cell clusters identified with FlowSOM. The cluster IDs and relative frequencies are displayed as a bar graph on the right-hand side, along with the median scaled expression. **b** UMAP plot displaying all cells from each FlowSOM-derived cluster identified in (**a**) coloured by cluster. **c** Schematic outline of UMAP identifying the main B-cell population as islands on the two-dimensional UMAP plot. Spatial differences between islands reflect the phenotypic differences between the identified B-cell populations. Similar populations aggregate together.

CD3 + T cells and CD19 + B cells accounted for a mean of 54.4% and 5.3% of the total live cell population across all samples, respectively. CD19 + cells were taken forward for further downstream analyses. FlowSOM analyses were repeatedly performed for reliability and expression of B-cell clusters were visualised as a heatmap (Fig. 2a) with heterogeneity in marker level displayed at the single-cell level using UMAP (Fig. 2b).

We defined 20 distinct B-cell clusters at various stages of maturation. The most frequently observed cluster was of the follicular B-cell lineage (cluster 5 (Fig. 2a–c)) characterised by high expression of CD20, CD22 and IgD and lower levels of IgM. Marginal zone (IgM + IgD^lo^ CD21^int^ CD27^lo^) (cluster 10) and transitional B-cell (IgM^hi^ CD24^hi^ CD38^hi^ CD5 + ) clusters (cluster 16) comprised 5.1% and 3.14% of the total B-cell population, respectively. Clusters 13 (0.89%) and 15 (2.16%) are also likely to represent transitional B-cell populations with high levels of CD10, CD24 and CD38. Both clusters are also high expressers of IgM and IgD, with cluster 13 being CD21^lo^ and cluster 15 being CD21^hi^. Memory B cells were observed at various stages of maturation: early memory B cells (cluster 19, 0.28%), fully affinity-matured, class-switched B cells (CD27hi IgD- IgM-) (cluster 14, 2.3%) and double-negative memory B cells (CD27lo IgD- IgM-) (cluster 11, 2.22%). We did not detect any clonal skewing of B-cell populations, measured through kappa/lambda mass cytometric assessment and baseline circulating kappa/lambda Ig ratio.

Seven suppressive B-cell clusters were identified based on canonical surface markers derived from the literature including CD5, CD24, CD25, CD27, CD38, CD1d, TIM-1, PD-1, PDL-1, TGF-β and/or the intracellular cytokine expression of IL-10. These were observed in varying frequencies (0.35–7.38%) (clusters 1, 2, 3, 9, 12, 17 and 18). Clusters 1, 3, 9, 12, 17 and 18 demonstrated low to intermediate IL-10 expression. Cluster 2 which is a PDL1 + cluster and although IL-10 negative is known to suppress via PD-L1 ligation independently of IL-10 and to express low levels of IL-10^19,24^. Since B cells were unstimulated IL-10 expression was representative of the in vivo peripheral immune milieu of NSCLC patients and healthy donors. Clusters 14 and 19 also express IL-10, however phenotypically they are much more likely to represent memory B cells; CD27 + IgD- IgM- affinity-matured, class-switched memory (cluster 14) and CD27 + IgD+ IgM− early memory (cluster 19). Similarly, cluster 20 (weakly IL-10-positive), phenotypically resembles a germinal centre B-cell population (IgM + IgD+ CD10 + CD27 + CD38 + BCL6 + ).

### B-cell phenotype associates with checkpoint blockade therapy irAEs

**Fig. 3:**
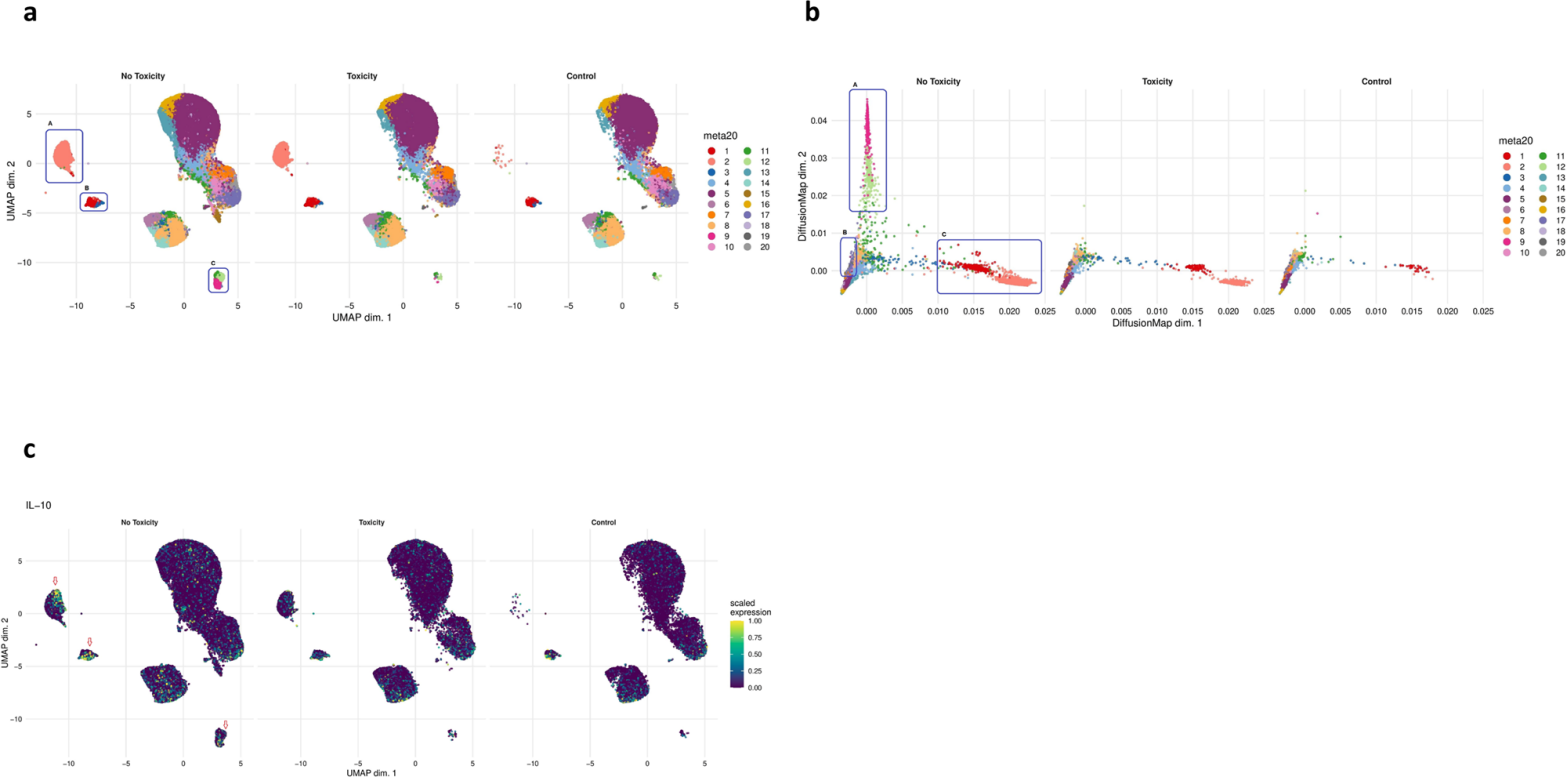
Comparative dimensionality reduction plots identify deficiencies of specific B-cell population in toxicity patients (*n* = 46). **a** UMAP plots stratified according to condition, “no toxicity”, “toxicity” and “healthy controls”. Clusters highlighted by blue boxes correspond to clusters 1, 2, 3, 9 and 12 identified from Fig. 2a. These clusters are attenuated in the “toxicity” cohort in this unsupervised analysis. The box labelled “A” is encircling cluster 2 (peach). The box labelled “B” is encircling clusters 1 (red) and 3 (blue). The box labelled “C” is encircling clusters 9 (pink) and 12 (light green). All samples are randomly downsampled to account for equally representative populations across samples. **b** Diffusion map stratified according to condition, “no toxicity”, “toxicity” and “healthy controls”. Clusters highlighted by blue boxes correspond to clusters 1, 2, 9, 12, 17 and 18 identified from Fig. 2a. These clusters are attenuated in the “toxicity” cohort in this unsupervised analysis. The box labelled “A” is encircling clusters 9 (pink) and 12 (light green). The box labelled “B” is encircling clusters 17 (deep lilac) and 18 (light lilac). The box labelled “C” is encircling clusters 1 (red) and 2 (peach). All samples are randomly downsampled to account for equally representative populations across samples. **c** UMAP plots stratified according to overall population IL-10 expression and by condition. Expression is largely confined to the Breg clusters demarcated by the red arrows. Matched IL-10 expression in the corresponding clusters in the toxicity patients is reduced. Although cluster 2 (peach island in (**a**)) is IL-10-negative in the heatmap, this is based on median marker expression and therefore does not illustrate the cell-to-cell variability in functional IL-10 status. The left-hand most red arrow depicts cluster 2 with subpopulations of IL-10 expressing cells (areas of light green and yellow), said cells are not seen in the corresponding cells in the toxicity patients.

Comparative analyses of B-cell subsets revealed striking differences in the frequencies of specific cell clusters between toxicity patients (*n* = 8), NT patients (*n* = 38) and healthy controls (*n* = 5) (Fig. 3a, b and Supplementary Fig. 4). The UMAP data clearly shows loss of clusters 9 and 12 together with loss of cluster 15 and significant attenuation of clusters 1, 2 and 3 in the toxicity patients. The diffusion map also very clearly shows loss of clusters 2 and 9, significant attenuation of clusters 1, 2 and 3 and loss of clusters 17 and 18. The loss of these latter two clusters is not immediately obvious on the UMAP as they are small subsets and lost in the group of similarly differentiated subsets. Thus, the proportions of the seven suppressive B-cell clusters (clusters 1, 2, 3, 9, 12, 17 and 18) were severely attenuated in the toxicity cohort relative to the NT cohort. On deeper interrogation of the clusters attenuated in the toxicity cohort (toxicity-linked clusters; TLCs), the most notable feature was IL-10 expression seen in clusters 1, 3, 9, 12, 17 and 18. In total, there are ten IL-10+ clusters in the entire B-cell population (with all the other 10 B-cell clusters being completely negative for IL-10 expression), with the expression of IL-10 residing within six out of the seven of the suppressive clusters of interest (clusters 1, 3, 9, 12, 17 and 18). Cluster 2 was clearly attenuated. Even though this is not an IL-10+ population, this is a PDL1^hi^ B-cell subset which has been described as suppressing via PDL1 independently of IL-10^19^. Whilst there is a presence of IL-10 in the TLCs in the NT patient group (Fig. 3c, TLCs demarcated by red arrows with IL-10-positive regions shown as yellow) there is clearly less IL-10 expression in the corresponding TLCs in the toxicity patients.

Interrogation of these suppressive clusters indicated strong phenotypic alignment to established Breg populations described in the literature^14,19,25–28^. Segregation of clusters 1 and 2 from clusters 9 and 12 (Fig. 3b) suggests the phenotypic differentiation of these groups is different. Plasmablasts and PDL1 + Bregs are immature^14,29^ suggesting earlier differentiation of these subsets with the acquisition of PD-1 and B10 markers reflecting late-stage maturation. Notably, only three clusters of the entire 20 B-cell subsets expressed PD-1/PDL1: cluster 9 was PD-1 + , and both clusters 2 and 18 were PDL1 + (Fig. 3b). The attenuation of the PD-1+ cluster^9^ in the toxicity cohort at diagnosis was particularly striking. In addition, cluster 18 uniquely expressed high levels of TGF-β, a known immunosuppressive cytokine. Of the remaining four clusters, we observed high CD5 expression as well as expression of CD24, CD25 and CD27 in clusters 12 and 17, and high CD38 and CD27 expression in clusters 1 and 3. Thus, phenotypically these seven attenuated clusters were characterised as (Fig. 3a):

A. Ki67^hi^ CD38^hi^ CD27^hi^ CD95^hi^ IL-10^int^ (cluster 1)
B. PDL1^hi^ CD38^int^ CD95^int^ TGF-β^-ve^ IL-10^-ve^ (cluster 2)
C. Ki67^int^ CD38^int^ CD27^int^ CD95^hi^ IL-10^lo^ (cluster 3)
D. PD-1^hi^ CD5^hi^ CD25^hi^ CD27^hi^ CD24^lo^ CD38^lo^ IL-10^int^ (cluster 9)
E. CD5^hi^ CD24^lo^ CD25^lo^ CD27^lo^ IL-10^lo^ (cluster 12)
F. CD24^hi^ CD25^hi^ CD27^hi^ IL-10^lo^ (cluster 17)
G. PDL1^hi^ CD38^int^ CD95^lo^ TGFβ^hi^ IL-10^lo^ (cluster 18).

Comparative median surface marker expression was conducted (Supplementary Fig. 5) and demonstrated higher median expression of canonical Breg markers in non-toxicity patients; CD25, CD5, CD27, CD24 as well as PD-1 PD-L1 and IL-10.

### Differential abundance analysis

**Fig. 4:**
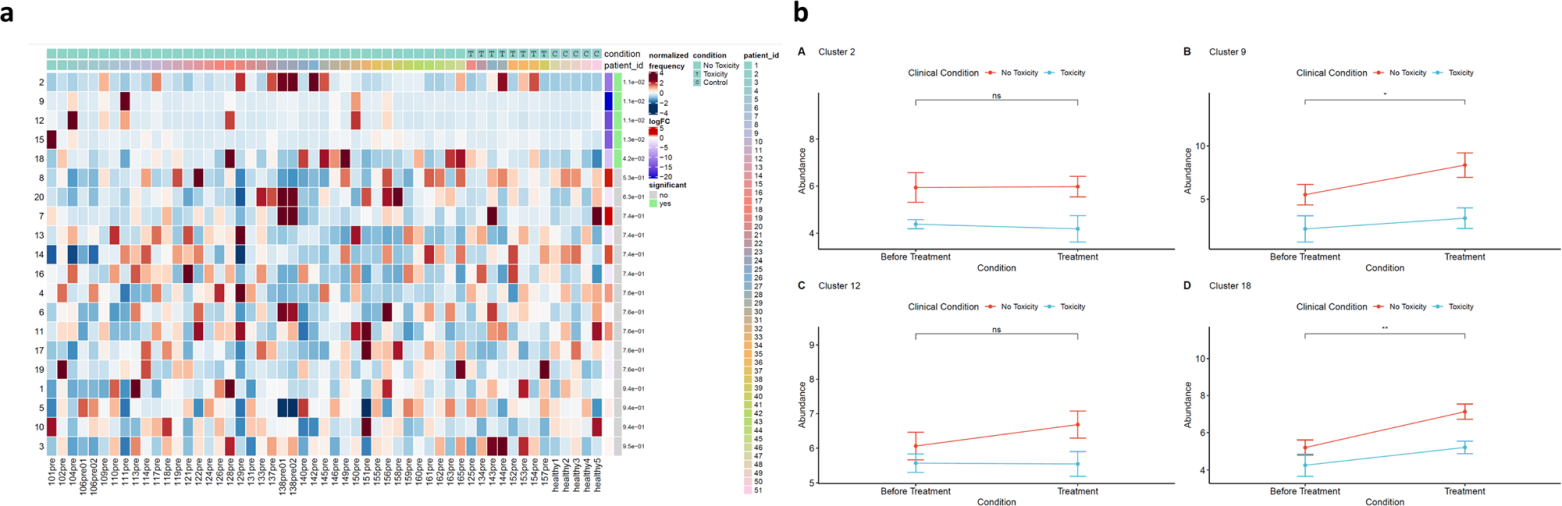
Differentially abundant B-cell populations between toxicity and non-toxicity patients. **a** Differential abundance heatmap illustrating 20 previously identified clusters (2A) (left-hand column) with a relative normalised abundance of each cluster by individual patient and healthy control sample (main panel). Patient to patient variability was treated as a random effect in order to improve the robustness of the model. A generalised linear mixed regression model was applied to determine the significance of differential abundance between conditions (toxicity and non-toxicity); the top five clusters were of statistical significance as shown by the green bars. Of these, four are the identified Breg populations. PDL1^hi^ CD38^int^ CD95^int^ TGFβ^-ve^ IL-10^-ve^ Breg (*P* = 0.011) (cluster 2 from initial unsupervised analysis), PD-1^hi^ CD5^hi^ CD25^hi^ CD27^hi^ CD24^lo^ CD38^lo^ IL-10^int^ Breg (*P* = 0.011) (cluster 9 from initial unsupervised analysis), CD5^hi^ CD24^lo^ CD25^lo^ CD27^lo^ IL-10^lo^ Breg (*P* = 0.011) (cluster 12 from initial unsupervised analysis) and PDL1^hi^ CD38^int^ CD95^lo^ TGFβ^hi^ IL-10^lo^ Breg (*P* = 0.042) (cluster 18 from initial unsupervised analysis). Cluster 15, an identified Transitional B-cell population (IgM^hi^ CD5- CD21^hi^ CD10^hi^ CD24^hi^ CD38^hi^) was also significantly more abundant in non-toxicity patients (*P* = 0.013). **b** Longitudinal analyses demonstrate significant increases in circulating Breg number post-treatment with checkpoint blockade therapy. Line plots indicate the fold change in circulating Breg cluster abundance before and after treatment with sub-stratification according to those who developed high-grade irAE after treatment (*n* = 19 biologically independent samples). Changes are shown for four major Breg phenotypes identified in the initial unsupervised analysis. None of the patients who developed toxicity post-treatment patients experienced significant increases in the circulating population number. The *P* value indicators are for non-toxicity patients. PDL1^hi^ CD38^int^ CD95^int^ TGF-β^-ve^ IL-10^lo^(2) [*P* = 0.952]; PD-1^hi^ CD5^hi^ CD25^hi^ CD27^hi^ CD24^lo^ CD38^lo^ IL-10^int^(9) [*P* = 0.035]; CD5^hi^ CD24^lo^ CD25^lo^ CD27^lo^ IL-10^lo^(12) [*P* = 0.104]; PDL1^hi^ CD38^int^ CD95^lo^ TGFβ^hi^ IL-10^lo^(18) [*P* = 0.009]. Data are presented as mean values + /− SEM. Statistical significance was determined by two-tailed pairwise Wilcoxon signed-rank test (significance level *P* < 0.05). *P < 0.05, **P < 0.01, ***P < 0.001. Source data are provided as a Source Data file.
- PD-L1^hi^ CD38^int^ CD95^lo^ TGFβ^hi^ IL-10^lo^ Breg (*P* = 0.042) (cluster 18).

We performed a differential abundance (DA) analysis of the defined cell populations, reporting on all B-cell clusters in the population (Fig. 4a). This allows comparison of the proportions of cell types between the three clinical conditions (toxicity, NT and healthy controls) and highlights the populations that are present at significantly different ratios. In order to gain the power to detect differences between conditions, we utilised a mixed model to model the response and patients were treated as a random effect thus formally accounting for the patient to patient variability as described by Nowicka et al.^30,31^. DA analysis of the overall cell population identified five clusters as significantly more abundant in the non-toxicity cohort as shown by the green bars in Fig. 4a. Four of these are the described suppressive B-cell populations (clusters 2, 9, 12 and 18 from initial unsupervised analysis) as follows:

- PD-L1^hi^ CD38^int^ CD95^int^ TGF-β^-ve^ IL-10^-ve^ Breg (*P* = 0.011) (cluster 2)
- PD-1^hi^ CD5^hi^ CD25^hi^ CD27^hi^ CD24^lo^ CD38^lo^ IL-10^int^ Breg (*P* = 0.011) (cluster 9)
- CD5^hi^ CD24^lo^ CD25^lo^ CD27^lo^ IL-10^lo^ Breg (*P* = 0.011) (cluster 12)

Cluster 15 was also significantly lower in the toxicity cohort and represents a transitional B-cell population and the relevance of this subset will be further considered below (Table 1).

**Table 1:** An overview of the observed toxicity-linked clusters in both independent cohorts.

Cellular phenotype	Cluster number (initial cohort)	Cluster number (validation cohort)	Biological relevance
PD-1^hi^	**9**—PD-1^hi^ CD5^hi^ CD25^hi^ CD27^hi^ CD24^lo^ CD38^lo^ IL-10^int^	**12**—CD5^lo^ **20**—CD5^hi^	The presence of PD-1^hi^ Bregs has been previously described in hepatocellular carcinoma (HCC) and correlated with disease progression and plasma IL-10 levels^25^. These exhibited a CD5^hi^ CD24^−/+^ CD27^hi/+^ CD38^dim^ phenotype. Incubation with HCC cell-derived supernatants induced B-cell PD-1 expression via TLR-4 ligation which when triggered induced IL-10 production and accelerated tumour progression. PD-1 + B cells from thyroid cancer patients suppressed T-cell proliferation and decreased T-cell viability, effects which could be reversed with PD-L1 blockade^41^. Patients with oesophageal squamous cell carcinoma had significant elevation of peripheral CD19 + CD24^hi^ CD27 + B10 cells with elevated peripheral B-cell IL-10 production^42^. B cells could internalise exosomes and treatment with exosomes from these patients could significantly increase the B10 population and stimulate both IL-10 and PD-1 expression, an effect apparently mediated via TLR-4 and MAPK pathway activation.
PDL1^hi^	**2**—PDL1^hi^ CD38^int^ CD95^int^ TGF-β^-ve^ IL-10^-ve^	Not observed	In the 4T1 breast cancer model, PD-L1 + B cells suppressed T-cell proliferation and were increased in the bone marrow, peripheral blood and spleen^44^. These PD-L1 + CD19 + cells were also upregulated in the peripheral blood of breast cancer patients^44^. Myeloid-derived suppressor cells (MDSCs) appeared to co-localise with marginal zone B cells, suggesting the potential for crosstalk between these two cell types within the tumour microenvironment. Indeed, co-culture of murine MDSCs with B cells upregulated PD-L1 expression and drove the B cells to become T-cell suppressive^44^.
	**18**—PDL1^hi^ CD38^int^ CD95^lo^ TGFβ^hi^ IL-10^lo^	**4**	
Plasmablast	**1**—Ki67^hi^ CD38^hi^ CD27^hi^ CD95^hi^ IL-10^int^	**16**	Groups have shown that the main Breg subset supressing myelin oligodendrocyte glycoprotein (MOG)-induced EAE are plasmablasts which regulate the generation of MOG-specific Th1 and Th17 cells by suppressing dendritic cells through high production of IL-10^14^. It was suggested that the protective effect of splenic B cells was due to plasmablast differentiation in draining lymph nodes: CD1d^hi^ CD5 + B10 cells differentiate into plasmablasts in culture^67^.
	**3**—Ki67^int^ CD38^int^ CD27^int^ CD95^hi^ IL-10^lo^	**17**	
B10-type	**12**—CD5^hi^ CD24^lo^ CD25^lo^ CD27^lo^ IL-10^lo^	**7**	B10 cells are characterised as CD1d^hi^ CD5 + ^26^. Whilst IL-10 expression is the best marker of human B10 plus B10 progenitor cells^27^, these cells form a subset of CD24^hi^ CD27 + B cells^27^ and both of these markers were present on cluster 17, with the expression of CD24 being the highest in all of the strong IL-10 expressing cells in this cluster. B-cell depletion significantly enhanced the local reaction to applied antigen in a contact hypersensitivity model where inflammation is T-cell-dependent^26^, and this was due to the significant reduction of a splenic IL-10-producing CD1d^hi^ CD5 + B-cell subset—B10 cells. Suppression of T-cell-driven inflammation was IL-10-dependent and in this model, IL-10 production was restricted to this particular B-cell subset. Adoptive transfer of CD1d^hi^ CD5 + B cells into CD19^−/−^ mice inhibited hypersensitivity responses but the transfer of CD1d^hi^ CD5 + B cells from unsensitised mice or from mice sensitised with an unrelated antigen had no effect, suggesting that these B10 cells were likely to be antigen-specific. B-cell depletion prior to EAE induction with MOG significantly exacerbated the resulting disease^68^. B-cell depletion prior to immunisation led to significant increases in MOG-specific T-effector cells and IFN-γ and IL-17 producing CD4 + cells. Adoptive transfer of modest numbers of B10 cells completely reversed the accelerated disease of B-cell-depleted mice prior to MOG immunisation. B10 function required IL-21R signalling, CD40 interaction and MHC class II expression^69^. A model was proposed whereby B cells capture autoantigen, thus triggering B-cell receptor (BCR) signalling and driving IL-10-producing capacity. These B cells present antigen via MHC class II in a cognate interaction to CD4 + T cells, which produce local IL-21, which can induce B-cell IL-10 production and subsequent T-cell suppression.
	**17**—CD24^hi^ CD25^hi^ CD27^hi^ IL-10^lo^	**8**	
Transitional (IgM + IgD+ CD5 + CD21 + CD10 + CD24 + CD38 + )	**13**—CD21^lo^	**1**—CD21^lo^	Transitional B cells are an intermediate population along the developmental axis, in between immature cells in the bone marrow and mature B cells in the spleen. In humans, they are characterised by high expression of CD10, CD24 and CD38^70^, but in reality are a heterogeneous population of cells with T1, T2 and T3 subsets having been identified with differences in surface marker expression of IgD, CD10, CD24 and CD38^71^. Moreover, as these cells differentiate further towards mature cells, there is a gradual loss of CD5, CD10 and IgM and upregulation of CD21 and CD22^72^. Functionally, the considerable overlap has been described between these cells and CD24^hi^ CD38^hi^ Bregs^29,32^. A CD27 + transitional B-cell subset has been described in humans which produces IL-10 in vivo and suppresses CD4 + T-cell proliferation and Th1 effector responses^32^. Cluster 15 was significantly deficient in toxicity patients in our cohort (*P* = 0.013).
	**15**—CD21^hi^ CD27 +	**2**—CD21^hi^	
	**16**—CD5 +	**6**—CD5 +	

The cohort of patients with low abundance of these specific clusters had a greater risk of high-grade irAEs. The rates of freedom from high-grade irAEs at 6 months were 100% (high) versus 58% (low) for the PD-1^hi^ subset (cluster 9) (*P* = 0.0061); for the PD-L1^hi^ TGF-β^-ve^ subset (cluster 2), 96% (high) versus 61% (low) (*P* = 0.0912); for the CD5^hi^ CD24^lo^ CD25^lo^ CD27^lo^ IL-10^lo^ (cluster 12), 98% (high) versus 73% (low) (*P* = 0.0038) and 89% (high) versus 55% (low) for the PDL1^hi^ TGF-β^hi^ subset (cluster 18) (*P* = 0.0387 Fisher’s exact test).

### Longitudinal analysis of B-cell changes on treatment

We then assessed the longitudinal effect of immunotherapy (pre-treatment and post cycle 1 (21-day interval)) on these four suppressive populations (Fig. 4b). In the NT patient group, treatment induced a significant increase in circulating PD-1^hi^ (cluster 9) and PD-L1^hi^ TGF-β^hi^ Bregs (cluster 18) with no change in the circulating PD-L1^hi^ TGF-β^-ve^ (cluster 2) or CD5^hi^ CD24^lo^ CD25^lo^ CD27^lo^ IL-10^lo^ (cluster 12) populations. However, in those patients who experienced severe toxicity, there was no significant increase in any of these subsets and the level of the PD-L1^hi^ TGF-β^-ve^ and the B10-type populations decreased.

### Breg/Tfh panel subgroup analysis

**Fig. 5:**
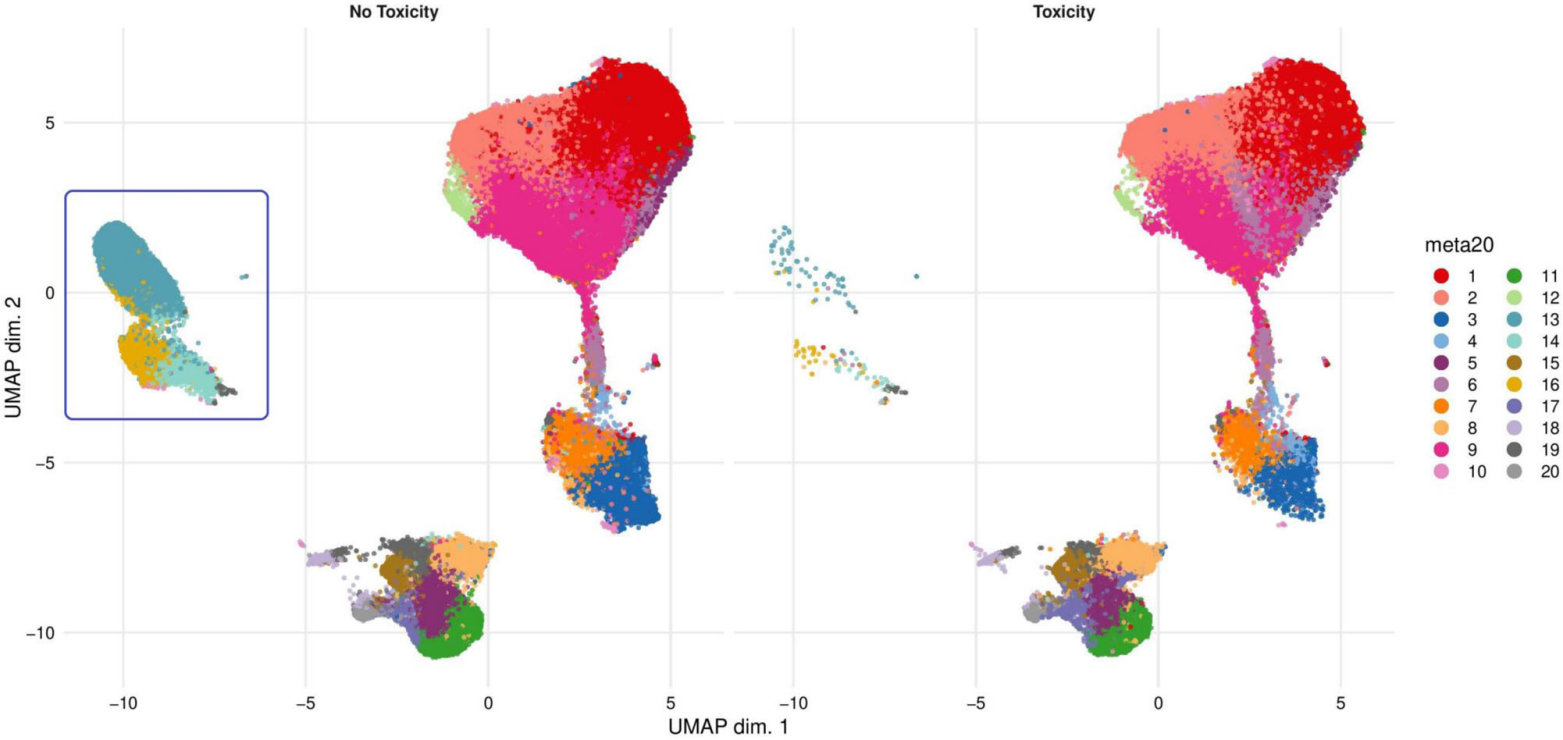
Comparative analysis of B-cell populations between toxicity and non-toxicity cohorts with Breg/Tfh panel (panel 2). UMAP plots stratified according to condition, “no toxicity” and “toxicity”. Clusters highlighted by blue boxes correspond to the most visually attenuated clusters in the “toxicity cohort” in this sub-analysis. These clusters are B10-type and PD-1 + in nature thus phenotypically similar to those identified in the previous analysis. All samples are randomly downsampled to account for equally representative populations across samples.

We analysed a randomly selected subgroup (from the initial cohort) of eight pre-treatment patients (five non-toxicity, three toxicity) using a more targeted Breg-specific panel. This panel also included markers for T follicular helper (T_FH_) and regulatory (T_FR_) cell lineages. The data were analysed as above using dimensionality reduction plots to display clusters by condition (Fig. 5) and sample (Supplementary Fig. 6). Clusters 13, 14 and 16 (all IL-10-positive) were all significantly deficient in toxicity patients based on DA testing (*P* < 0.01). Clusters 13 and 16 are both PD-1 + , with cluster 13 being PD-1^int^ and cluster 16 being PD-1^lo^. Both clusters also express TIGIT and CD1d, with higher levels of both in cluster 13. Cluster 14 is a B10-type cluster. Importantly, it is a significant lack of all three types of clusters, which seems to be the consistent feature amongst the toxicity patients identified (Fig. 5).

### B-cell phenotyping of an independent cohort of NSCLC patients using mass cytometry

**Fig. 6:**
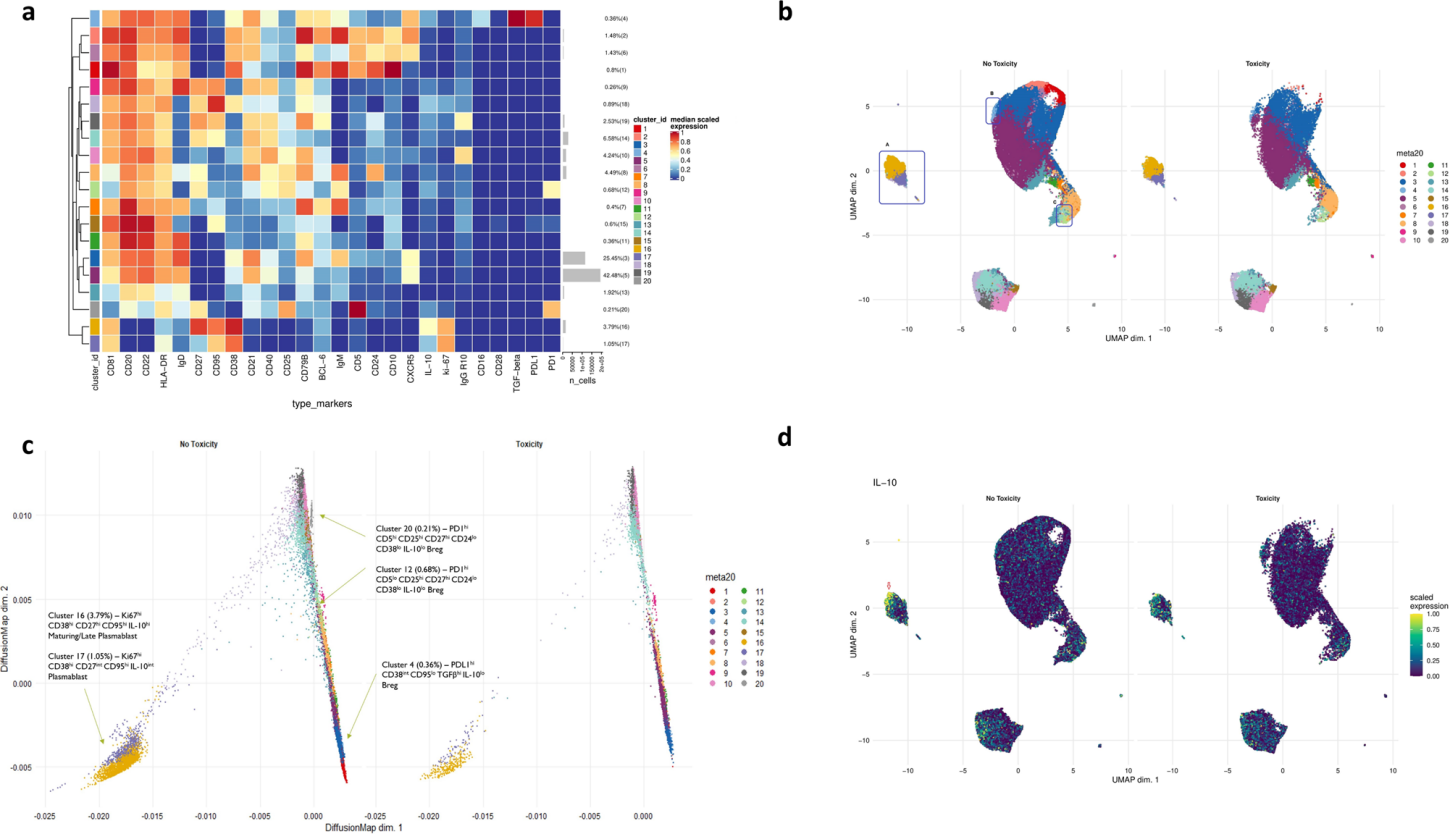
Deficiencies of specific B-cell populations in toxicity patients (*n* = 8) identified in an independent validation NSCLC cohort. **a** Heatmap showing normalised expression of the base B-cell panel markers for the 20 B-cell clusters identified with FlowSOM. The cluster IDs and relative frequencies are displayed as a bar graph on the right-hand side, along with the median scaled expression. **b** UMAP plots stratified according to condition, “no toxicity”, “toxicity” and “healthy controls”. Clusters highlighted by blue boxes correspond to clusters identified from (**A**). These clusters are attenuated in the “toxicity” cohort in this unsupervised analysis. The box labelled “A” is encircling clusters 16 (mustard) and 17 (lilac). The box labelled “B” is encircling cluster 4 (light blue). The box labelled “C” is encircling cluster 12 (light green). All samples are randomly downsampled to account for equally representative populations across samples. **c** Diffusion map stratified according to condition, “no toxicity”, “toxicity” and “healthy controls”. Clusters labelled 4, 12, 16, 17 and 20 are attenuated in the “toxicity” cohort in this unsupervised analysis. Cluster 4 is shown by the thin “light blue” line of cells to the right of the darker blue cells in cluster 3. All samples are randomly downsampled to account for equally representative populations across samples. **d** Bulk expression UMAP stratified according to IL-10 expression, which demonstrates less intense IL-10 expression in the toxicity cohort compared to the non-toxicity patients. Expression is largely confined to the Breg clusters demarcated by the red arrows. Matched IL-10 expression in the corresponding clusters in the toxicity patients is reduced.

Finally, we validated our findings using an entirely separate cohort of pre-treatment lung cancer patients, treated with anti-PD-1 therapy, from the PAIR study (REC reference 17/LO/1950), in which the CyTOF analysis was performed independently using an identical experimental and analytical pipeline. This was a small but well-dichotomised group of patients (*n* = 8) with two of the patients experiencing high-grade toxicity on checkpoint blockade therapy. Using the first B-cell mass cytometry panel, we created a comprehensive overview of the B-cell landscape (Fig. 6a).

Comparative expression with UMAP (Fig. 6b) shows a striking loss of clusters 1 and 2 in the toxicity cohort. These map to transitional cell clusters 13 (CD21^lo^) and 15 (CD21^hi^ CD27 + ) respectively from our initial cohort, which were also deficient in toxicity patients; cluster 15 was significantly attenuated in toxicity patients on differential abundance testing (*P* = 0.013). Cluster 4 was significantly attenuated in toxicity patients (Fig. 6b) and this maps to the similarly deficient PDL1^hi^ CD38^int^ CD95^lo^ TGFβ^hi^ IL-10^lo^ population, cluster 18 from our initial cohort. The diffusion map (Fig. 6c) shows a clear loss of clusters 12 and 20 (PD-1^hi^) and a significant attenuation of clusters 16 and 17 (plasmablast) in the toxicity cohort. Clusters 12 and 20 map to cluster 9 (PD-1^hi^) from our initial cohort. Finally, clusters 16 and 17 map to clusters 1 and 3, respectively, in our initial cohort, thus providing validation of our initial findings.

In-depth phenotyping of this independent cohort, identified these clusters of interest (clusters 4, 12, 16, 17 and 20) as follows:

- PD-L1^hi^ CD38^int^ CD95^lo^ TGFβ^hi^ IL-10^lo^ (cluster 4)
- PD-1^hi^ CD5^lo^ CD25^hi^ CD27^hi^ CD24^lo^ CD38^lo^ IL-10^lo^ (cluster 12)
- Ki67^hi^ CD38^hi^ CD27^hi^ CD95^hi^ IL-10^hi^ (cluster 16)
- Ki67^hi^ CD38^hi^ CD27^int^ CD95^hi^ IL-10^int^ (cluster 17)
- PD-1^hi^ CD5^hi^ CD25^hi^ CD27^hi^ CD24^lo^ CD38^lo^ IL-10^lo^ (cluster 20).

These clusters are highlighted in Table 1 (below) with parallels made to clusters identified in the initial cohort. In this cohort, we noted two PD-1+ populations largely delineated from one another by levels of CD5 expression^25^ and two plasmablast populations with varying degrees of proliferation (ki67 positivity) and IL-10 expression. Cluster 16 was the most strongly IL-10-positive population. The PD-L1 + population (cluster 4) displayed high levels of PD-L1 expression, which has been observed in the original description of this heterogeneous population^19^. Similar to our original NSCLC cohort, we noted that clusters 9, 10, 14, 18 and 19 were also IL-10-positive but phenotypically were representative of the memory B-cell spectrum. Clusters 14, 18 and 19 are CD27 + IgD− IgM− affinity-matured, class-switched memory cells, cluster 9 is CD27 + IgD+ IgM− early memory and cluster 10 is likely to represent a double-negative memory B cell. All these clusters co-localise on UMAP reinforcing their phenotypic similarities.

Bulk expression of IL-10 (Fig. 6d) shows predominant expression focused to the suppressive B-cell clusters of interest (notably clusters 12, 16 and 17). Despite the presence of these suppressive phenotypes in toxicity patients (albeit at lower levels compared to non-toxicity patients), there is the diminished expression of IL-10 in these populations in toxicity patients. Figure 6b also shows a striking loss of clusters 1, 2 and 6 in toxicity patients: phenotypically these are likely to represent IgM+ IgD+ CD5 + CD21 + CD10 + CD24 + CD38 + transitional B cells which represent B cells at a key stage in their developmental pathway and encompass a range of transitional B-cell subsets with unique regulatory functional profiles including those which suppress via IL-10^32^. These three clusters (1, 2 and 6) mirror the transitional cell populations, clusters 13, 15 and 16 defined in our initial NSCLC cohort, respectively. In the initial cohort, cluster 15 was one of the 5 significantly reduced B-cell clusters in toxicity patients. Cluster 1 in this validation cohort and cluster 13 from the original are both CD21^lo^ which is known to be associated with high PD-1 expression.

To the best of our knowledge, these data provide the first detailed analysis of the association of circulating IL-10-positive Bregs with the subsequent development of severe irAE following immunotherapy in NSCLC patients. Patients developing grade 3/4 irAEs have a significant functional and phenotypic defect in their regulatory B-cell repertoire prior to commencing immunotherapy, which does not appear to replenish on treatment with checkpoint blockade. Our primary analysis was focused on analysing the relationship between B-cell function and repertoire and immune-related adverse events, however, we also looked at the relationship to response to checkpoint blockade treatment.

### B-cell phenotype associates with response to checkpoint blockade therapy

**Fig. 7:**
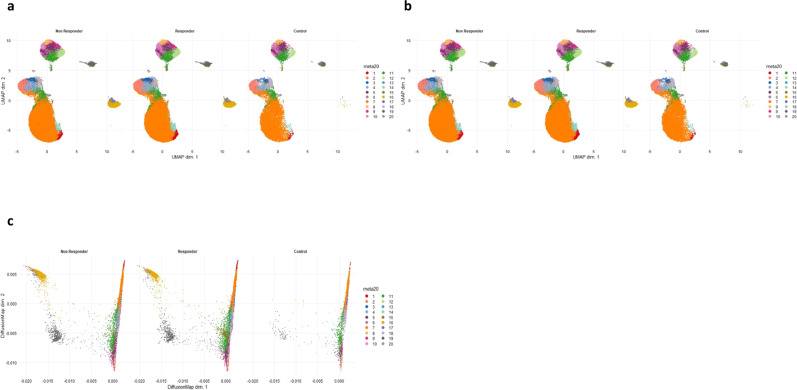
Comparative dimensionality reduction plots show no major reciprocal deficiencies of toxicity-linked clusters in non-responders (*n* = 46). **a** UMAP plots stratified according to condition, “responder”, “non-responder” and “healthy controls”. There are no major visual differences in cluster abundance between responders and non-responders. This is also the case when specifically looking at the toxicity-linked clusters. All samples are randomly downsampled to account for equally representative populations across samples. **b** Diffusion map stratified according to condition, “responder”, “non-responder” and “healthy controls”. There are no major visual differences in cluster abundance between responders and non-responders. This is also the case when specifically looking at the toxicity-linked clusters. All samples are randomly downsampled to account for equally representative populations across samples. **c** UMAP plots stratified according to overall population IL-10 and PD-L1 expression and by condition. Expression is largely confined to the suppressive B-cell clusters demarcated by the red arrows, however, there is no major difference in IL-10 expression between conditions, but there is higher expression of PD-L1 in non-responders. Both PDL1 + clusters (green box) were significantly more abundant in non-responders when analysed with differential abundance analysis (*P* < 0.05, Benjamini–Hochberg correction).

We performed comparative analyses between responders (*n* = 26) and non-responders (*n* = 20) using dimensionality reduction methods (Fig. 7a, b). There were no striking differences in cluster proportion between responders and non-responders. When stratified according to IL-10, there was no significant difference in expression in the suppressive clusters between cohorts. However, PDL-1 expression in clusters 16 and 17 was noticeably higher in non-responders (Fig. 7c). These two clusters phenotypically map to clusters 2 and 18 from the toxicity analysis, respectively.

A. PDL1^hi^ TGFβ IL-10^-ve^ (cluster 16)

B. PDL1^hi^ TGFβ^hi^ IL-10^lo^ (cluster 17)

Differential abundance analysis (Supplementary Fig. 7) indicated significantly higher expression of the immature suppressive PDL1 phenotypes; 16 and 17 in non-responders (*P* = 0.002 and *P* = 0.00022, respectively).

We interrogated a publicly available mass cytometry dataset in melanoma^33^ and gated the files specifically for CD19 + positive cells and utilised the panel’s B-cell markers to cluster the cells. The cohort was split into those who received Pembrolizumab (anti-PD-1, *n* = 40) or Ipilimumab (anti-CTLA-4, *n* = 24) as first-line therapy. Each of these two cohorts had a stimulated and an unstimulated fraction. In the unstimulated fraction, we were able to detect the same PDL1 + populations accounting for the limitations due to panel differences. Differential abundance revealed a significantly higher proportion of PD-1 + IL-10 + suppressive B cells in non-responders, which phenotypically mapped to cluster 9 from our toxicity analysis (*P* = 0.00069). From our dataset, aside from the PDL1 + cells, no further link with TLCs and response to treatment was observed. The relationship between toxicity and response to treatment was not significant (*P* = 0.121 by Chi-Squared with Yates’ correction).

## Discussion

As the use of checkpoint inhibitors in cancer has increased, the incidence of irAEs has become a significant health care issue. Bregs can suppress immune reactivity against exogenous and endogenous antigens and in particular they can act as a cellular brake on the development of autoimmunity, often via IL-10 production^12^. We are aware of only one study that analyses the role of B cells in the development of irAEs secondary to checkpoint blockade^34^. On-treatment changes in peripheral B cells were analysed in 39 melanoma patients, 23 of whom were treated with combination immunotherapy. A B-cell parameter comprising a >70% decline in B-cell number and a >twofold increase in either CD21^lo^ B cells or plasmablasts was reported. None of the patients with B-cell changes treated with combination therapy were free of grade 3 or greater irAEs at 6 months compared with 87% of those without such B-cell changes, and such patients were more likely to develop multi-organ autoimmune toxicities^34^.

Through testing in independent cohorts, we show here that lung cancer patients developing severe toxicity from anti-PD-1/PD-L1 blockade had significant gaps in their B-cell profile at diagnosis, specifically affecting suppressive B-cell subsets (IL-10 + , TGF-β + or PDL-1 + ). All subsets detected in this context display strong phenotypic parallels with previously described Breg subsets. Patients who did not subsequently develop severe toxicity had preservation of these subsets at diagnosis and in these patients, the levels of these cells increased on checkpoint blockade whereas in toxicity patients Breg populations they either remained static or fell. Our phenotyping data suggests there is an attenuated presence of circulating Breg populations in toxicity patients and our functional data suggests that the B cells in toxicity patients not only fail to mount an adequate anti-inflammatory cytokine (IL-10) response but also an adequate pro-inflammatory cytokine (IFN-γ, IL-6, IL-17) response. Pro-inflammatory cytokine production has been demonstrated by B cells ex vivo and in the setting of checkpoint blockade in melanoma^35,36^. TLR-9 ligation upregulates B-cell IL-6 production and PMA/ionomycin drives IFNγ production^37^. Both of these components are part of the Breg IL-10 stimulation cocktail and neither IL-10, IL-6 or IFNγ production was seen in B cells from patients developing toxicity. B-cell-specific IFN-γ production has been identified as a mechanism underlying the pathological germinal centre formation and auto-antibody production via T_FH_ cell expansion^38^. IFNγR signalling in B cells is required for spontaneous germinal centre formation and T_FH_ cell development in non-autoimmune B6 mice. IFNγR and STAT1 signalling promotes the germinal centre formation and the expansion of T_FH_ cells via expression of the transcription factor T-bet and IFNγ production by B cells^39,40^. This pan-cytokine failure in B cells from toxicity patients may explain the lack of a reciprocal relationship between toxicity and response to treatment. The nature and mechanistic underpinning of this polyfunctional defect and which implies B-cell exhaustion in toxicity patients is unknown and is a key part of our ongoing studies.

We describe the loss of several well defined Breg subsets in toxicity patients. The presence of PD-1^hi^ Bregs (cluster 9 (PD-1^hi^ CD5^hi^ CD25^hi^ CD27^hi^ CD24^lo^ CD38^lo^ IL-10^int^) in initial cohort, and clusters 12 and 20 in validation cohort, (Table 1)) has been previously described in several cancers^25,41,42^, where they have been shown to be known producers of IL-10 and positively correlate with disease progression^25^. PD-1 triggering and PD-L1/PD-1 ligation induced IL-10 production and CD5^hi^ PD-1^hi^ B cells were major IL-10 producers. This PD-1-driven IL-10 production suppressed T-cell immunity, thus accelerating tumour progression^25^, and this suppressive function may be reversible with checkpoint blockade.

PD-L1^hi^ Bregs (clusters 2 (PDL1^hi^ CD38^int^ CD95^int^ TGF-β^-ve^ IL-10^-ve^) and 18 (PDL1^hi^ CD38^int^ CD95^lo^ TGFβ^hi^ IL-10^lo^) in the initial cohort and cluster 4 in the validation cohort, (Table 1)) limit the expansion of the humoral immune response in part by blocking the differentiation of T_FH_ cells^19^. T_FH_ cells typically express PD-1 and interact with B cells in the B-cell follicle to initiate the germinal centre reaction, whereby B cells undergo expansion and affinity maturation. Elevated levels of T_FH_ cells are well-described in autoimmune diseases, characterised by auto-antibody formation, particularly in rheumatic diseases^43^. PD-L1^hi^ B cells have been found to express significantly less IL-10 compared with PD-L1^lo/int^ B cells and moreover suppress the T-cell response independently of IL-10^19^. This PD-L1 dependent IL-10-independent T-cell suppression has been demonstrated in breast cancer^44,45^. The abundance of this specific B-cell phenotype in non-responders supports the impact of PD-L1 ligation on T-cell suppression. Autoimmune thyroiditis is a common irAE to checkpoint inhibitors. The percentage of CXCR5 + circulating T_FH_ cells was elevated in patients with Hashimoto’s thyroiditis compared with controls, as were the percentages of PD-1+ T_FH_ cells^46^. The percentage of PD-1+ T_FH_ cells positively correlated with anti-thyroglobulin antibody levels and negatively correlated with FT_3_ levels. PD-L1 blockade of PD-L1 + Bregs from untreated rheumatoid arthritis (RA) patients increased the proliferation and cytokine production of CD8 + T cells^47^. These PD-L1 + Bregs were significantly decreased in untreated patients compared with healthy controls and were increased upon successful treatment of rheumatoid arthritis.

The highly proliferative plasmablast-like subsets (clusters 1 (Ki67^hi^ CD38^hi^ CD27^hi^ CD95^hi^ IL-10^int^) and 3 (Ki67^int^ CD38^int^ CD27^int^ CD95^hi^ IL-10^lo^) in initial cohort and 16 and 17, respectively, in validation cohort, Table 1)) subsets have been described with close phenotypic similarity to our observed subsets (CD27 + CD38 + IL-10 + )^14^. Their suppressive capabilities through IL-10 production has been shown to be protective in murine models of EAE^14^. CD19 + CD24^hi^ CD38^hi^ transitional B cells have been shown to be functionally defective in patients with relapsing and remitting multiple sclerosis resulting in a failure to suppress effector T-cell responses and promoting autoimmunity^48^. This lack of transitional subsets has also been noted as contributory to the unregulated autoimmune processes in other neurological disorders such as neuromyelitis optica^49^ (Table 1).

Three patients (total of 8 toxicity) developed severe colitis, one of the most serious autoimmune complications of checkpoint blockade therapy. In the Dextran sodium sulphate (DSS) model of ulcerative colitis (UC), CD19^−/−^ mice were more susceptible to DSS-induced intestinal damage, accompanied by greater intestinal infiltration of neutrophils and T cells^50^. The proportion of splenic B cells was inversely proportional to the degree of intestinal damage and to the proportion of circulating B10 cells increased during DSS-induced damage. The administration of IL-10 to CD19^−/−^ mice significantly decreased intestinal damage and adoptive transfer of B10 cells to CD19^−/−^ mice significantly reduced colitis. Co-administration of peritoneal IL-10 competent B cells also reduced the severity of colitis induced by the intraperitoneal administration of CD25^-^CD45RB^hi^ CD4 + T cells^51^. CpG-induced B-cell production of IL-10 and the frequency of IL-10-producing B cells (CD19^hi^ CD1d^hi^) was significantly reduced in patients with Crohn’s disease (CD) or UC, compared with B cells from healthy donors^10^. In humans, B10 + and B10pro cells are a subset of CD24^hi^ and CD27 + B cells.

Compelling evidence shows that Bregs directly suppress autoreactive T cells, suppress T_FH_ cell generation thereby limiting autoreactive B cells, and thus serve as a brake on autoimmunity^19,28^. Thus, it is entirely plausible that a defective Breg brake could predispose to the development of autoimmunity upon the use of checkpoint blockade. A deficient Breg checkpoint is likely to fail to limit the enhanced activity of self-reactive T cells and auto-antibody formation, which are enabled by PD-1/PD-L1 blockade. It is also possible that anti-PD-1 treatment will also directly reduce the suppressive functions of PD-1+ and PD-L1 + Bregs. Consistent with this scenario, we show here that patients who subsequently develop toxicity following checkpoint blockade treatment have a clear defect in their functional B-cell repertoire at diagnosis, with a significant reduction of separate IL-10 expressing B-cell subsets, which closely map to Breg subsets described in the literature.

Finally, Tregs are capable of suppressing inflammatory responses through the formation of tolerogenic antigen-presenting cells (APCs) via IL-10 and TGF-β, which then upregulate PD-L1^52^. Studies have shown that in diseases such as multiple sclerosis^53^, there is a loss of functional suppression by CD4 + CD25 + Tregs despite circulating presence and in conditions such as SLE^54^, both a functional and proportional loss of circulating Tregs has been demonstrated. Our data has not demonstrated a significant difference in CD4 + CD25 + Treg abundance between toxicity and NT patients with lung cancer.

The high-dimensional nature of the data allied with the analysis facilitated robust exploration, biomarker discovery, annotation, and differential abundance analysis. However, the technique and analysis will be limited by manual gating methods, which are open to subjectivity, bias towards well-known subtypes and inefficiency in larger datasets. The development of integrated machine learning methods will help to bridge the gap with other OMICS data analyses and help to infer developmental trajectories directly from cytometry data^55^. Although many irAEs occur early, longer-term follow-up will be required to ensure that the NT patients remain free of severe irAEs. Furthermore, we restricted our analysis to lung cancer patients treated with PD-1/PD-L1 blockade. Such analyses will need to be performed in patients with other cancers and in those treated with anti-CTLA-4 checkpoint blockade.

**Fig. 8:**
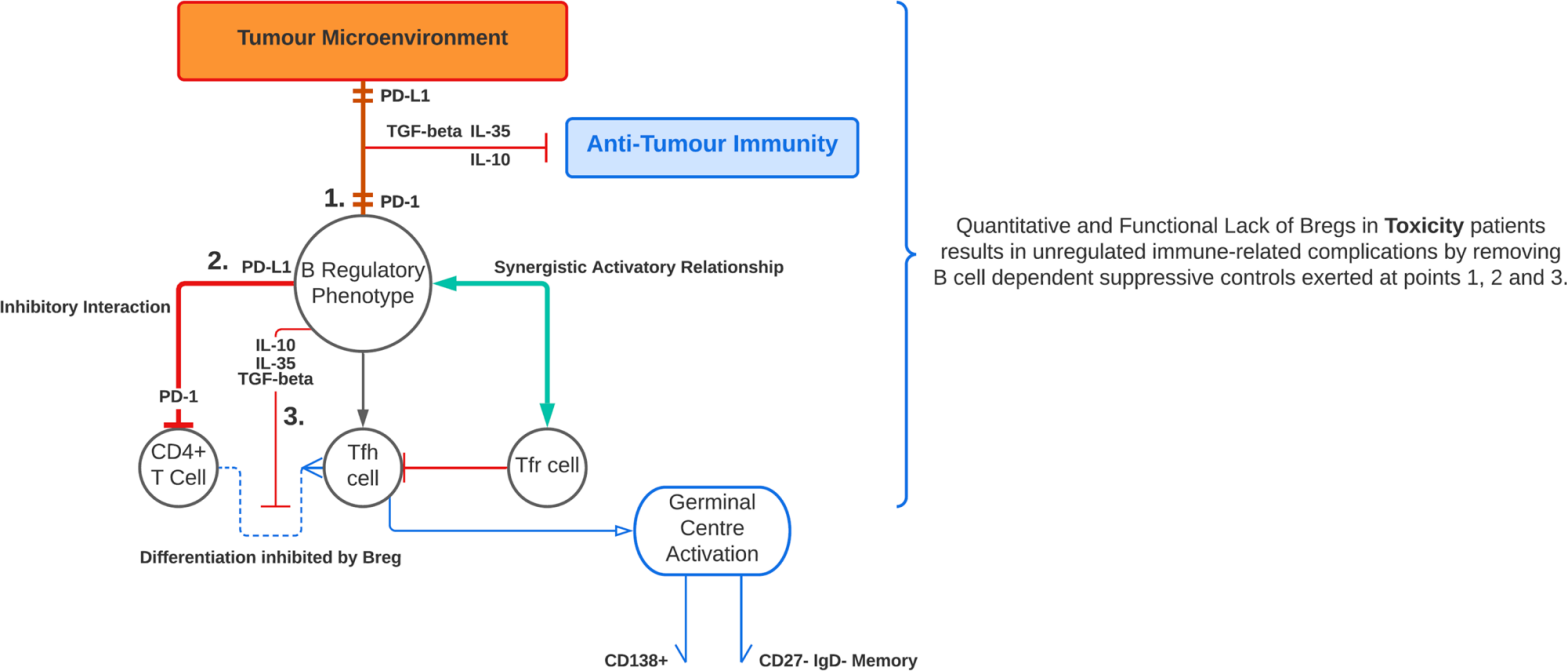
A schematic demonstrating Breg dynamics and suppressive mechanisms between toxicity and non-toxicity patients. This schematic illustrates the interplay between Bregs, CD4 + T cells, Tfh and Tfr cells and the role of suppressive cytokines and the PD-1-PD-L1 axis in mediating downstream responses. Bregs can be PD-1 or PDL1-positive. Interactions with tumour cell PD-L1 (1) or upon encountering PD-L1 + cells, this PD-1 triggering results in the acquisition of suppressive functions with tumour-specific T-cell immunity and promotes cancer growth via IL-10 production. Interaction with CD4 + specific PD-1 via B-cell-specific PD-L1 (2) inhibits Tfh maturation and differentiation from CD4 + T cells and subsequent entry into the germinal centre where Tfh help induces B-cell maturation into memory and terminally differentiated plasma cells. These direct PD-1-PD-L1 interactions between CD4 + and Breg cells, respectively, result in Stat5 upregulation and subsequent Tfh suppression. CXCR5 + CD25 + Foxp3 + T follicular regulatory cells work synergistically with Breg cells to skew the balance away from Tfh maturation and expansion. Tfh suppression also occurs via suppressive cytokine signalling (3). These three mechanisms are thought to be the B-cell-exerted brakes on autoimmunity which prevents unregulated immune-related adverse events following checkpoint blockade.

In summary, we show that patients who have inherent functional defects in their Breg repertoire, as well as deficits in specific peripheral Breg phenotypes, are predisposed to developing severe autoimmunity (Fig. 8). Our findings, despite the small size of the external validation cohort, significantly enhance our understanding of the aetiopathogenesis of severe autoimmunity under checkpoint blockade. They have significant implications for our ability both to predict the likelihood of severe immune toxicity with checkpoint blockade, and ultimately also to develop tolerable checkpoint blockade regimens in such high-risk patients. Future translational applications are both potentially diagnostic and therapeutic. Narrower spectrum flow cytometry assays at diagnosis would help formulate an idea of the circulating Breg milieu in patients and when combined with functional IL-10 assays, would stratify the risk of toxicity in patients undergoing checkpoint blockade therapy. By stratifying susceptible patients based on Breg profile and comparing to those patients included for treatment according to standard criteria as parallel trial arms, we could gain useful insight into the utility of this type of cellular risk stratification. Similarly, developing a large prospective cohort of patients would inform us as to whether specific Breg signatures or deficiencies correlate with toxicity at anatomical sites. As was shown in murine models of intestinal damage, administering an IL-10 adjuvant either as a stable mRNA vaccine or via CD19 + adoptive B-cell transfer, to patients experiencing toxicity, tissue damage could be considerably limited. This could be a significant therapeutic application if not prophylactic in those who are deemed highly susceptible to toxicity.

## Methods

### Experimental model and participant details

Peripheral blood mononuclear cells (PBMC) from age-matched healthy donors were obtained from the Clinical Immunology Service at the University of Birmingham (UoB). Primary blood samples from advanced Non-Small Cell Lung Cancer (NSCLC) patients were obtained before and after the first cycle of treatment with checkpoint blockade immunotherapy either as monotherapy or in combination with chemotherapy. Written consent was provided under the UoB Research Ethics Approval, protocol 10/H0501/39. Tumour stage and histological subtype with molecular profiling was determined by a radiologist and pathologist respectively (Supplementary Table 1). The PAIR study was used to provide the validation arm samples, this was based at Guys and St Thomas’ NHS foundation trust. All uses of human material from this study was approved by the Guy’s and St. Thomas’ Research Ethics approval (REC reference 17/LO/1950) and written consent was provided by all participants recruited under this research approval.

### Sample preparation and acquisition

PBMCs were harvested by density gradient centrifugation using BD vacutainer^®^ CPT bottles (NH: ~130IU FICOLL^TM^ 2.0 ml). Following centrifugation, the cells were washed twice with RPMI-1640, and resuspended in freezing media (sterilised mix of 90% heat-inactivated foetal calf serum and 10% DMSO) at a density of 4–10 × 10^6^/ml prior to cryostorage at −80 °C.

### Cell staining for mass cytometric acquisition

CyTOF antibody cocktails (cell surface and intracellular done separately) were prepared using pre-determined optimal titres and filtered using Ultrafree MC 0.1-μm centrifugal filter units (Merck Millipore) to remove antibody aggregates. Cryopreserved cells were resuscitated for mass cytometry experiments by rapid thawing at 37 °C, slow dilution with wash media and then centrifugation to pellet cells and removal freezing media. The cells were then filtered through a 35-μm nylon mesh using 5-ml tubes with cell strainer caps and then washed with MaxPar Cell Staining Buffer (CSB, Fluidigm). Cells were then incubated with 5 µl of Fc receptor blocking reagent (Human Trustain Fc blocking solution, Biolegend) for 10 min at room temperature and then immediately incubated with surface antibodies at room temperature for 30 min. For post-treatment samples (anti-PD-1 therapy), PD-1 expression was detected by combining anti-IgG4-biotin (HP-6025; Sigma) with anti-PD-1 antibody (S2) and adapted for mass cytometry as previously described^56,57^. During the last 2 min of this incubation, cells were incubated with 1 µM cisplatin to allow live cell (cisplatin-) / dead cell (cisplatin + ) discrimination. The reaction was quenched with CSB (Fluidigm). Cells were then fixed and permeabilised for intracellular antibody staining using MaxPar Fix I Buffer (Fluidigm^®^) and MaxPar Perm-S Buffer (Fluidigm^®^) (two washes) respectively. Stimulation of cells prior to intracellular antibody was not performed to avoid altering rare cellular phenotypes and investigate constitutive expression reflective of the microenvironment^58,59^. The cells were resuspended and 2 µl of Heparin solution (2k U/ml stock) was added to each sample to prevent non-specific binding of charged eosinophils for a total of 10 min. The intracellular antibody cocktail was then added to the cells. After gentle agitation, the suspension was left to incubate for 30 min at room temperature. Cells were then washed with buffer and resuspended in 500 µl of Cell Intercalation Solution (1:1000 Nucleic acid Rh^103^ Intercalator: Fix and Perm Buffer (Fluidigm^®^)) and incubated overnight at 4 °C.

### Preparation for data acquisition

The next day, samples were washed twice with cell staining buffer, resuspended in 1 ml of MilliQ ddH_2_O, filtered through a 35-µm nylon mesh (5-ml tubes with cell strainer caps, BD) and counted. Before analysis, samples were resuspended in MilliQ ddH_2_O supplemented with EQ four-element calibration beads (Fluidigm^®^) at a concentration of 0.5–1.0 × 10^6^ cells/ml. Samples were acquired at 300 events per second on a Helios instrument (Fluidigm^®^) using the Helios 6.5.358 acquisition software (Fluidigm^®^). We collected a minimum range of 750,000–1.2 million cells per sample in order to maximise the chances of detecting rarer B-cell subsets. IL-10 detection albeit low in the unstimulated mass cytometry cohort, was reflective of likely Breg populations given the surface phenotype. We performed corroborative work on a stimulated cohort of melanoma cells which showed no difference in IL-10 when compared to the parallel unstimulated cohort^33^. Samples were acquired using two separate B-cell panels (Deep B-cell phenotyping and B-regulatory cell/T follicular helper cell panels). Individual.fcs files collected from each set of samples were concatenated using the.fcs concatenation tool from Fluidigm^®^ (CyTOF normalisation software 2), and data were normalised based on EQ four-element signal shift over time using the same tool.

### Antibody labelling and conjugation protocol

In-depth characterisation of B cells within our cohort was performed using metal-tagged antibodies. Metal conjugated antibodies were purchased from Fluidigm or conjugated to unlabelled antibodies in-house. All unlabelled antibodies were purchased in carrier-free form and conjugated with the corresponding metal tag using the MaxPAR antibody conjugation kit (Fluidigm^®^) as per the manufacturer’s instructions. Metal isotopes were acquired from Fluidigm. The concentration of each antibody was assessed after metal conjugation using a Nanodrop 2000 (ThermoFisher Scientific). Conjugated antibodies were diluted using PBS-based antibody stabiliser supplemented with 0.05% sodium azide (Sigma-Aldrich) to a final concentration of 200 µg/ml and were subsequently titrated to an optimal concentration for use. Provider, clone and metal tag of each antibody used in this study are provided in S2.

### Cell isolation and culture

PBMCs were isolated as described earlier. CD4 + T cells and CD19 + B cells were subsequently isolated by magnetic cell sorting, based on CD4 microbead positive selection and subsequent CD19-negative selection (MACS, Miltenyi Biotech); purity was consistently >98%. Cells were cultured in 10% FBS, RPMI-1640 (Sigma) with 2 mM l-glutamine (Sigma), 0.1 g/L sodium bicarbonate (Sigma), supplemented with 100 U/mL penicillin and 0.1 mg/mL Streptomycin (PAA), and 10 mM Hepes (PAA), in round-bottom 96-well Nunclon plates (Thermo Scientific) at 0.3 × 10^6^ cells/well. To induce IL-10 expression, the TLR-9 ligand CpG ODN 2006 (Miltenyi Biotech) was used, alongside IL-2 (250 mcg/ml, Aldesleukin, Peprotech). B cells were cultured for up to 40 h. This time point was chosen as most appropriate for the detection of IL-10, based on kinetics of expression. Cells were re-stimulated with PMA (1 mg/mL) and Ionomyocin (1 mg/mL, Sigma) in the presence of Brefeldin A, and GolgiStop (both 1 mg/ml) for three hours at a 1:1000 concentration, and then stained for intracellular cytokine production.

### T:B-cell co-culture

Autologous CD4 + T and CD19 + B cells were isolated as described above. CD4 + T cells were stimulated with plate-bound CD3 and CD28 (both 1 mg/ml, Biolegend). B cells were added to the T cells in a 1:1, 1:2 and 1:4 ratio and cultured for 4 days. After 91 h, cells were re-stimulated with PMA (1 mg/ml) and Ionomyocin (1 mg/mL, Sigma) in the presence of Brefeldin A, and GolgiStop (both 1 mg/ml) for 5 h, and then stained with fluorescent-labelled antibodies specific for intracellular cytokines.

### CD4 T-cell proliferation assay

To assess the effect of IL-10 on CD4 T-cell proliferation, cell trace violet (Thermofisher Scientific) was used as per manufacturer recommendation. CD4 + T cells isolated from PBMCs were stained with 5 µM cell trace violet (Thermofisher Scientific), following the manufacturer’s instructions. Cells were then cultured with varying concentrations of CD19 + B cells from 0.6 × 10^5^–0.24 × 10^6^/well. They were subsequently cultured in 96-well flat-bottom plates, containing plate-bound CD3 and CD28 (both 1 mg/ml), at 0.3 × 10^6^ cells per well. After 4 days, cells were harvested and acquired for staining and flow cytometry.

### Intracellular flow cytometry

Antibodies used, and their corresponding fluorophores, are shown in Supplementary Table 3. For cytokine detection, cells were re-stimulated in the final 3 (CD19 + only) or 5 h (co-culture) of culture with PMA (1 mg/mL) and Ionomyocin (1 mg/mL, Sigma) in the presence of Brefeldin A, and GolgiStop (both 1 mg/ml, both BD Biosciences). Cells were harvested and incubated with Live/Dead, then washed and fixed in 4% paraformaldehyde (Fixation Buffer, Biolegend) for 15 minutes at room temperature. Cells were then incubated with the appropriate volume of antibody, diluted in 0.1% Saponin in 0.5% BSA in PBS for 45 minutes at room temperature. Cells were acquired using a BD FACSCanto II (BD Biosciences), and analysis was conducted using MRC Cytobank software (Tree Star Inc.). Gating was carried out using either a fluorescence minus one control, and/or unstimulated cells (Supplementary Figs. 8 and 9).

### Quantification and data analysis

Files (.fcs) were processed and normalised as described and uploaded into Cytobank (Beckmann Coulter Inc.), populations of interest were manually gated, biaxial marker expression was performed for visualisation in Cytobank and events of interest were exported as.fcs files. CD19 + sample ‘clean-up’ was performed by gating on intact (103Rh + DNA stain), no beads (140Ce − ), live (194/195Pt − ), no T cells CD3 − (141Pr), no immature granulocytes or natural killer cells CD16 − (209Bi) and CD45 + (89Y), CD19 + B cells.

For downstream analysis,.fcs files were loaded into R (R Core Development Team, 2015, v4.0.3) using R Studio (v4.0.3). Signal intensities for each channel were arcsinh transformed with a cofactor of 5 (x_transf = asinh(x/5)). In order to facilitate differential discovery and analysis within our dataset, we employed a hybrid R-based pipeline largely based on Bioconductor packages; flowCore^60^, FlowSOM^23^, CATALYST^30^, diffCYT^61^.

High-resolution, unsupervised clustering and meta-clustering were performed using the FlowSOM and ConsensusClusterPlus packages respectively, the former allowed for scaling of millions of cells therefore no sub-sampling of the data was required^23,30^. Visualisation of data was carried out through the CATALYST package, which employs the ggplot2 R package as the graphical engine. To visualise the high-dimensional cell populations in two dimensions, the UMAP (Uniform Manifold Approximation and Projection) algorithm^22^ was applied in order to represent the characteristics of the annotated cell populations and identified biomarkers. Spectral non-linear dimension reduction was carried out using diffusion maps^62,63^ in order to follow the distribution of differentiating cells. Differential cell abundance analysis was performed using generalised linear mixed models (GLMM), implemented via the diffCYT package^30,61^ using a false discovery rate adjustment (at 5% using Benjamini–Hochberg method) for multiple hypothesis testing. In order to identify the main cell subsets using both B-cell panels, FlowSOM was run with the parameter *k* ((x dim = 10 x ydim = 10) = 100), defining the number of nearest neighbours, set to 100. The function then metaclustered populations into 2 through maxk (default 20) clusters^23^. In order to confirm and extend our biological discovery, the clustering algorithm (*k*-means) was modified to detect a maximum of eight meta-clusters after assessing the initial unsupervised 20 meta-clusters for biological relevance and this was to deduce which clusters were deemed most important according to the algorithm. This was performed following a review of the delta plot as detailed by Nowicka et al.^31^. Furthermore, selective marker clustering algorithms were run to assure us of true marker expression within the clusters of interest. In order to further define specific B-cell clusters, runs were carried out with Principal Component Analysis (PCA) pre-processing incorporating all markers on the panel (including those for T-cell lineage) and then run without these markers (namely CD3, CD4, CD8). This was done to exclude those that are not expressed on B cells and likely to add “noise” in the cluster generation process, so with the aim to increase the impact of the biologically relevant markers^64,65^.

Statistical significance was determined using a two-tailed non-parametric test for unpaired (Mann–Whitney *U* test) or paired (Wilcoxon rank-sums test) samples and for more than two independent groups, Kruskal–Wallis. Univariate and Multivariate Stepwise Backward Elimination models were also constructed. Boxplot visualisation was carried out using the ggplot2 visualisation engine through the ggpubr (v0.4.0) package^66^. The risk of high-grade irAE was determined using Fisher’s exact test. The time to toxicity was compared between low and high abundances of a particular cluster within the cohort using the log-rank method; this was carried out in R using the Survival and Survminer packages for Kaplan–Meier analysis and Cox-proportional hazards regression, respectively. Suitable data cutpoints were determined using the pROC and cutpointr R packages for ROC and bootstrap analyses respectively. Pairwise comparisons in longitudinal analyses were carried out using the pairwise Wilcoxon rank-sums test. A *P* value less than 0.05 was considered significant. Multiple comparisons correction was applied using the Benjamini–Hochberg method.

### Reporting summary

Further information on research design is available in the Nature Research Reporting Summary linked to this article.

## Supplementary information

Supplementary Information

Reporting Summary

## Data Availability

Mass and flow cytometry data: the data that support the findings of this study are available from the corresponding author upon reasonable request. This is largely owing to file size and logistics of patient confidentiality, reverse pseudonymisation and need for data to be kept at specific academic/research sites in line with the policies from individual trial protocols.  Source data are provided with this paper.

## Source data

Source Data

## Code availability

The authors declare that the code for reproducibility of data are publicly available. Although the code was adapted from various sources, the underlying code itself was not modified or changed in any way and is readily available from the sources cited. The code can be made available from the corresponding author upon reasonable request.
