# Supplementary material for: Regulatory B cell repertoire defects predispose lung cancer patients to immune-related toxicity following checkpoint blockade
Source: Nat Commun. 2022 Jun 7;13:3148. doi: 10.1038/s41467-022-30863-x (PMC9174492; doi:10.1038/s41467-022-30863-x)
Supplement: Supplementary file 1 — Supplementary Information [file 41467_2022_30863_MOESM1_ESM.pdf]

Supplementary Information

Supplementary Figure 1. Representative Flow plots for B cell only IL10

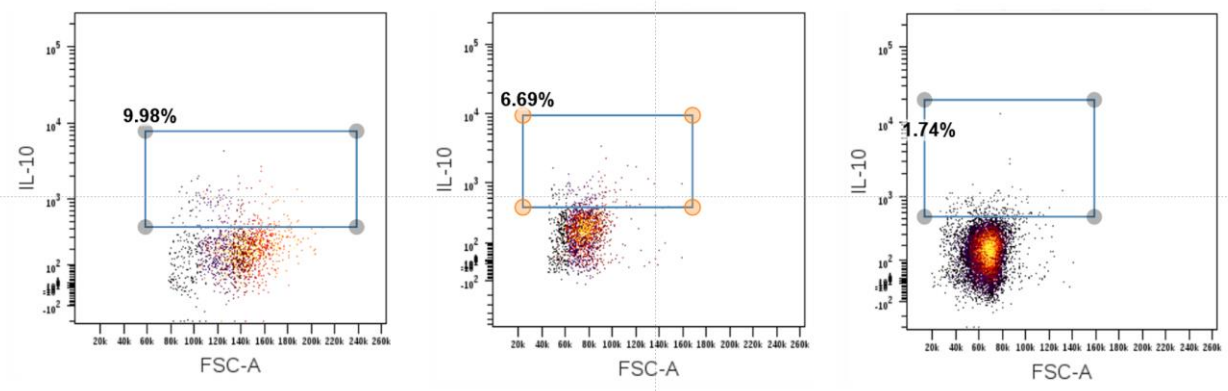

Healthy Control   Non-Toxicity Patient   Toxicity Patient

Supplementary Figure 2. IL-10 assay in cell culture supernates (ELISA 450nm)

CD19+ B cell IL-10 expression in NSCLC patients stratified by the development of high-grade post-treatment irAE. Data are presented as concentration (pg/ml) of IL-10 in cell culture supernates, with points indicating individual patients (n=45 biologically independent samples including controls). Box plots are defined as median (centre), with the bounds of the box representing the interquartile range (upper and lower bounds) and the whiskers representing upper and lower extremes. Statistical analysis was conducted using Wilcoxon Rank Sums test (to test for specific inter-group differences) and Kruskal Wallis (to generally test for overall differences between all three groups). All analyses were conducted using a Benjamini-Hochberg multiple comparisons correction, comparing all conditions as indicated. A significance level of <0.05 was adopted.

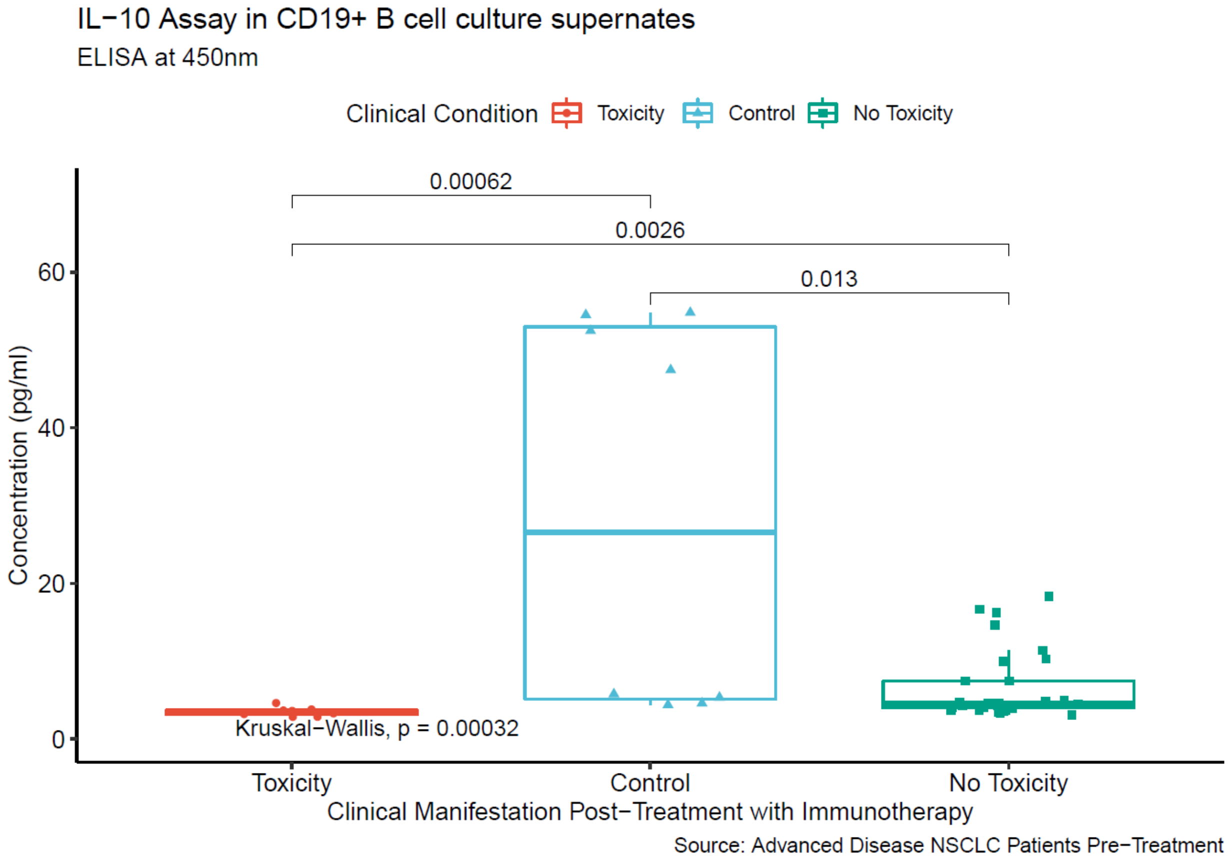

<u>Supplementary Figure 3. CD4+ T cell suppression flowplots</u>

No Toxicity

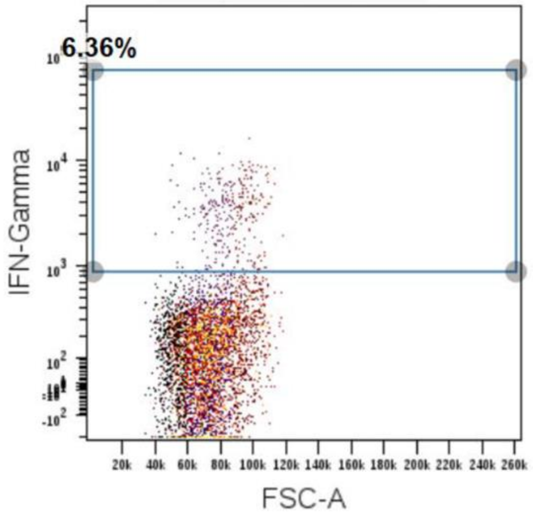

CD4+ T cell alone

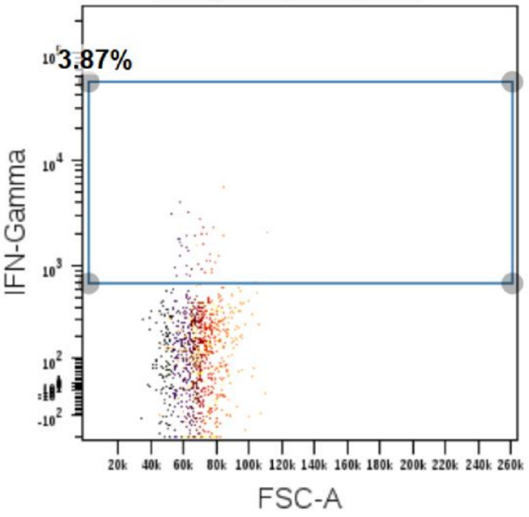

CD4:B cell co-culture (1:2)

Toxicity

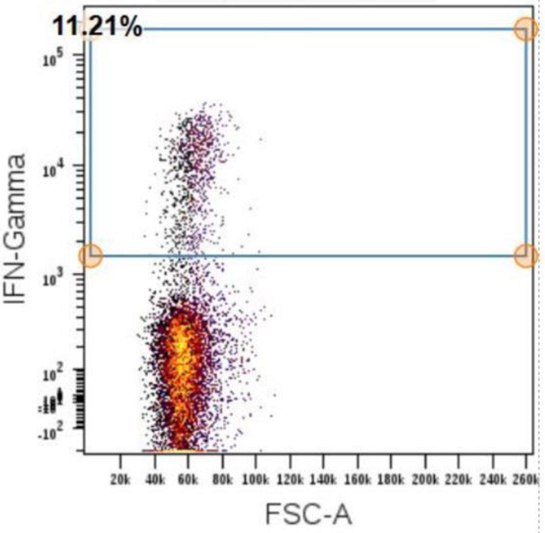

CD4+ T cell alone

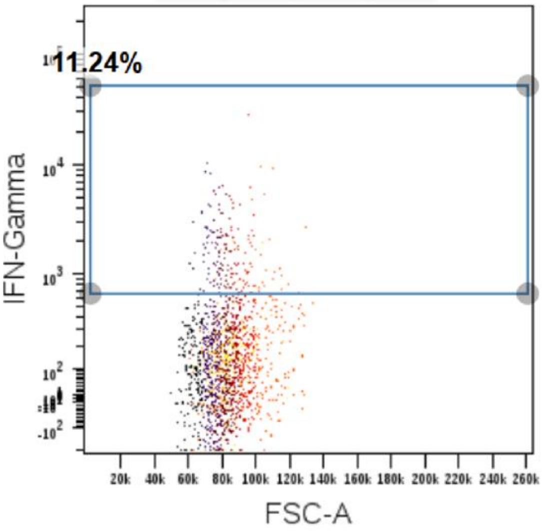

CD4:B cell co-culture (1:2)

Supplementary Figure 4. UMAP by sample (Initial Cohort)

UMAP plots stratified according to individual patients. UMAPS highlighted by the red box correspond to the toxicity patients. All samples are randomly downsampled to account for equally representative populations across samples.

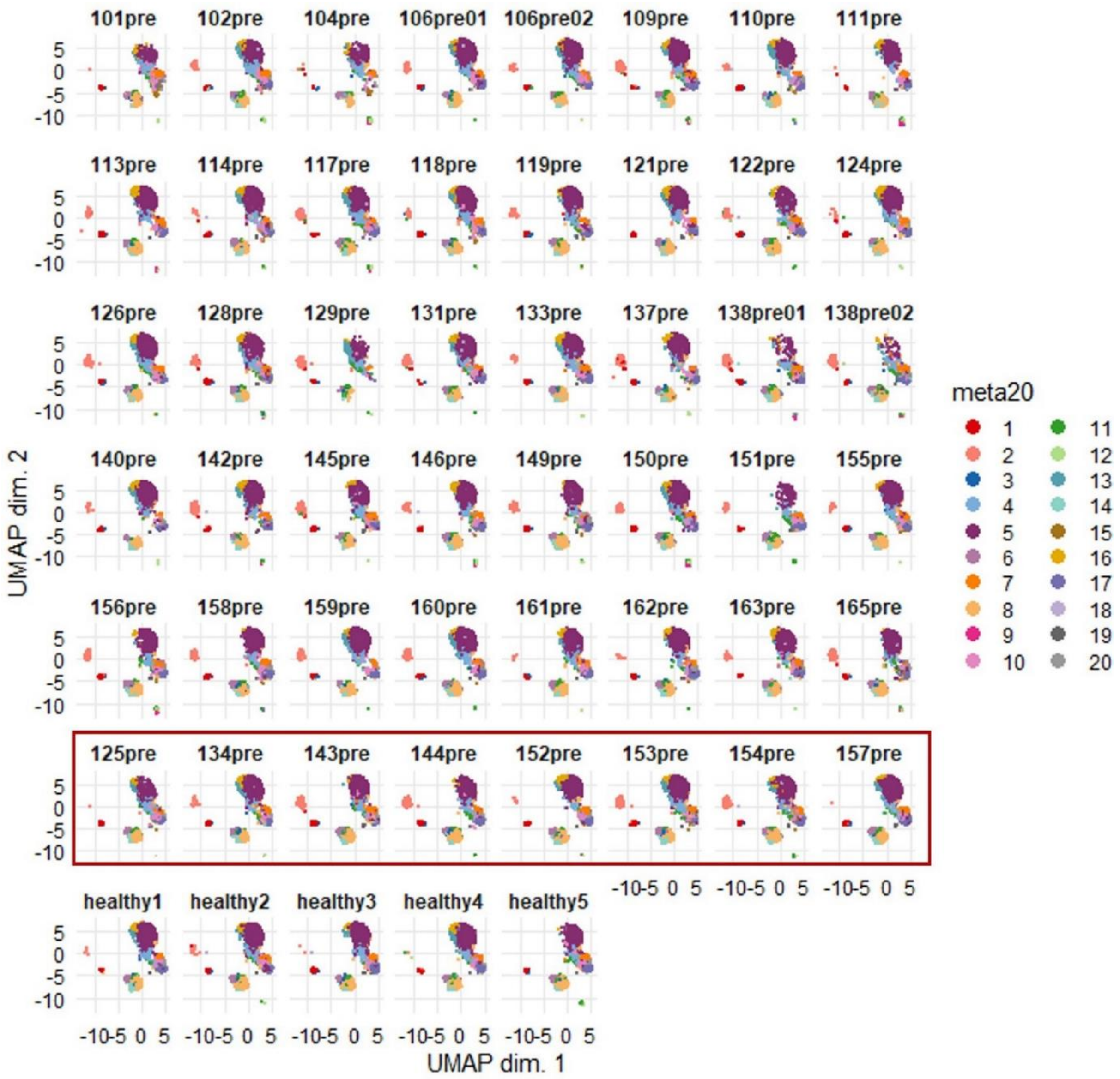

Supplementary Figure 5. Comparative Median Marker Expression by Condition (Initial Cohort)

Median marker expression in NSCLC patients stratified by the development of high-grade post-treatment irAE. Data are presented as median marker abundance using mass cytometric high dimensional analysis, with points indicating individual patients (n=53 biologically independent samples including controls). Box plots are defined as median (centre), with the bounds of the box representing the interquartile range (upper and lower bounds) and the whiskers representing upper and lower extremes.

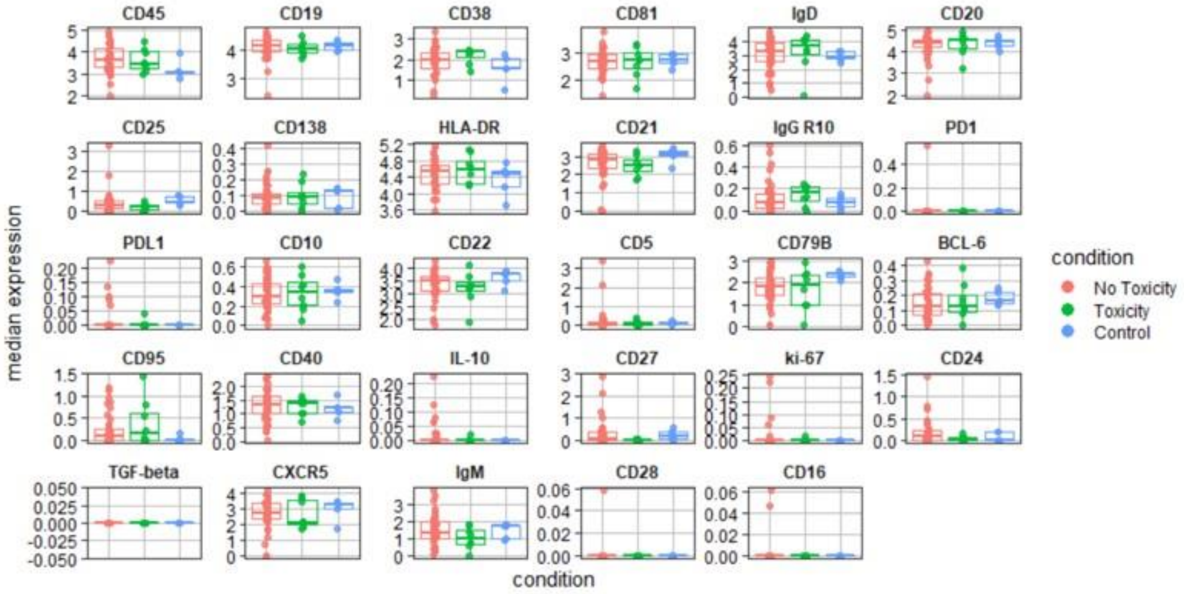

Supplementary Figure 6. UMAP by sample – Breg/Tfh panel subcohort

UMAP plots stratified according to individual patients using subgroup analysed with Breg/Tfh panel (n=8 biologically independent samples). UMAPS highlighted by the red boxes correspond to the toxicity patients. All samples are randomly downsampled to account for equally representative populations across samples.

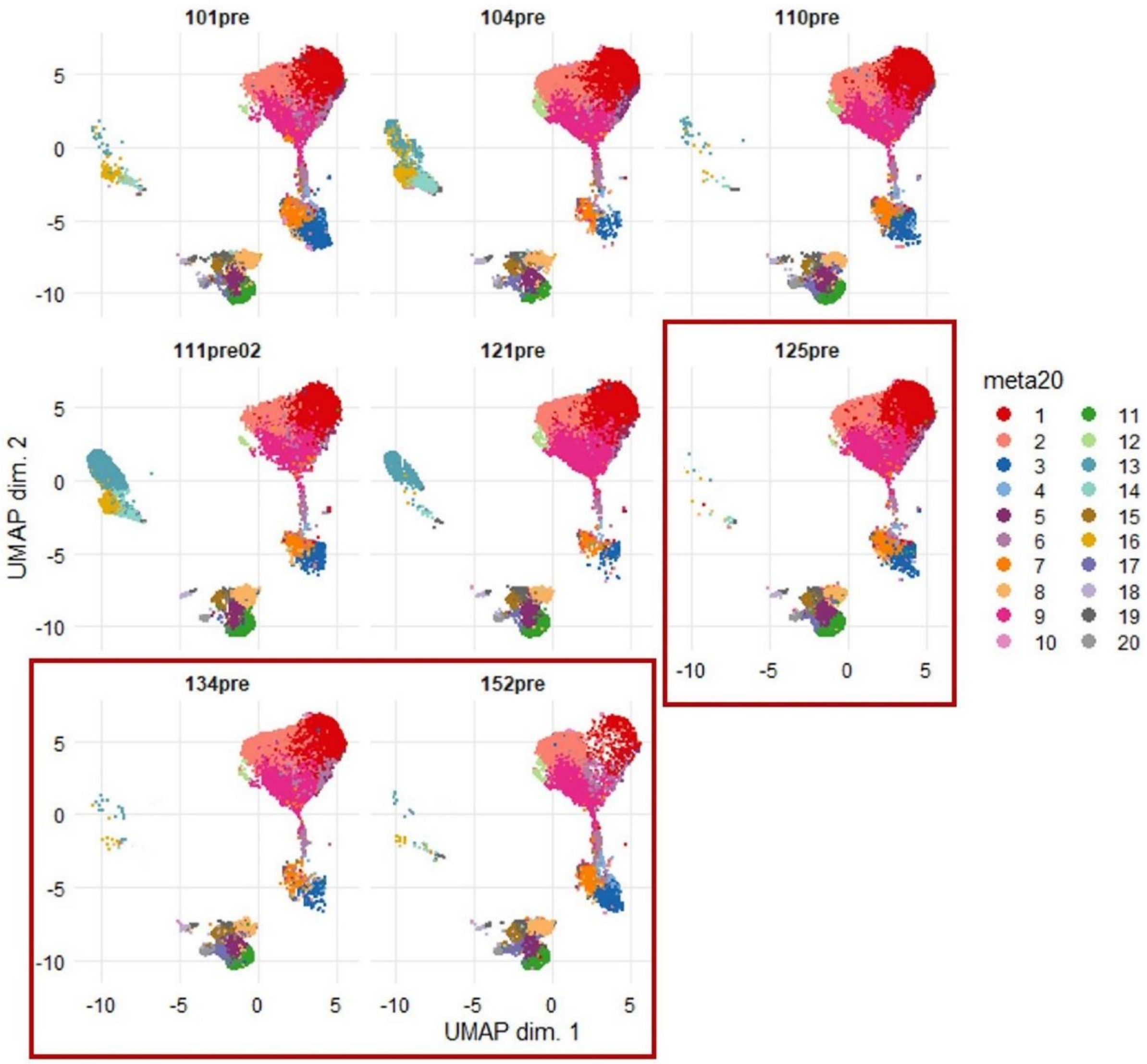

Supplementary Figure 7. Differential Abundance Analysis by Response

Differential Abundance Heatmap illustrating 20 previously identified clusters (2A) with relative normalised abundance of each cluster by individual patient and healthy control sample (main panel). Patient to patient variability was treated as a random effect in order to improve the robustness of the model. A generalised linear mixed regression model was applied to determine significance of differential abundance between conditions (responders and non-responders); the top two clusters were of statistical significance as shown by the grey bars.

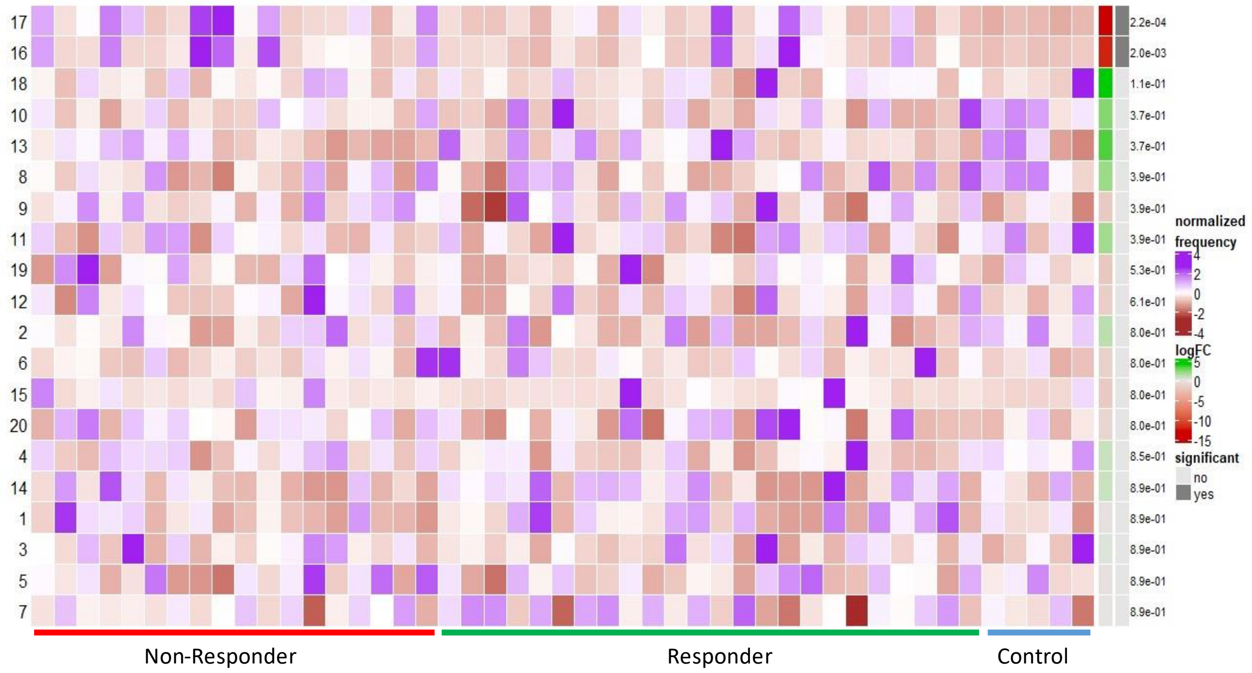

Supplementary Figure 8. Gating Strategy for Flow Cytometric analyses of IL-10 expression in B cells

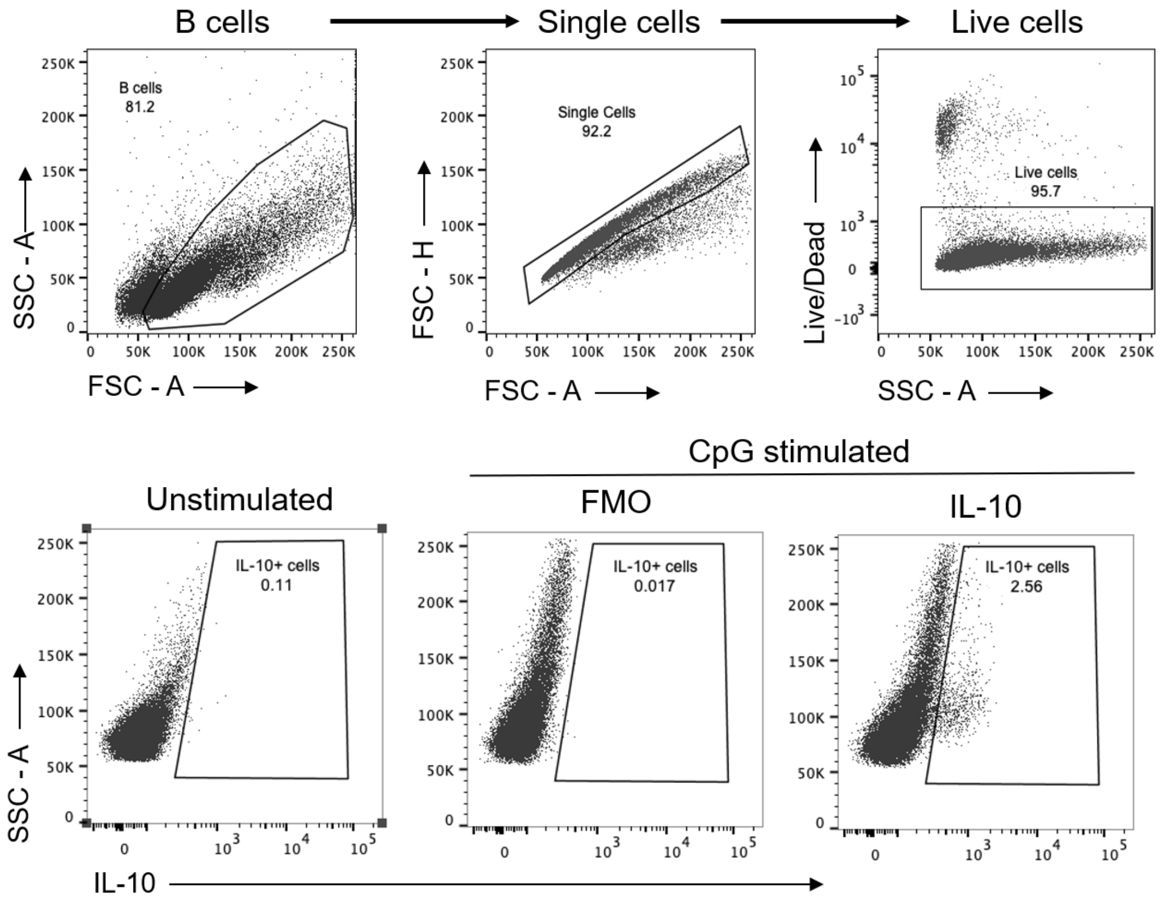

Supplementary Figure 9. Gating Strategy for Flow Cytometric analyses of IFNy expression in CD4+ T cells upon co-culture with B cells

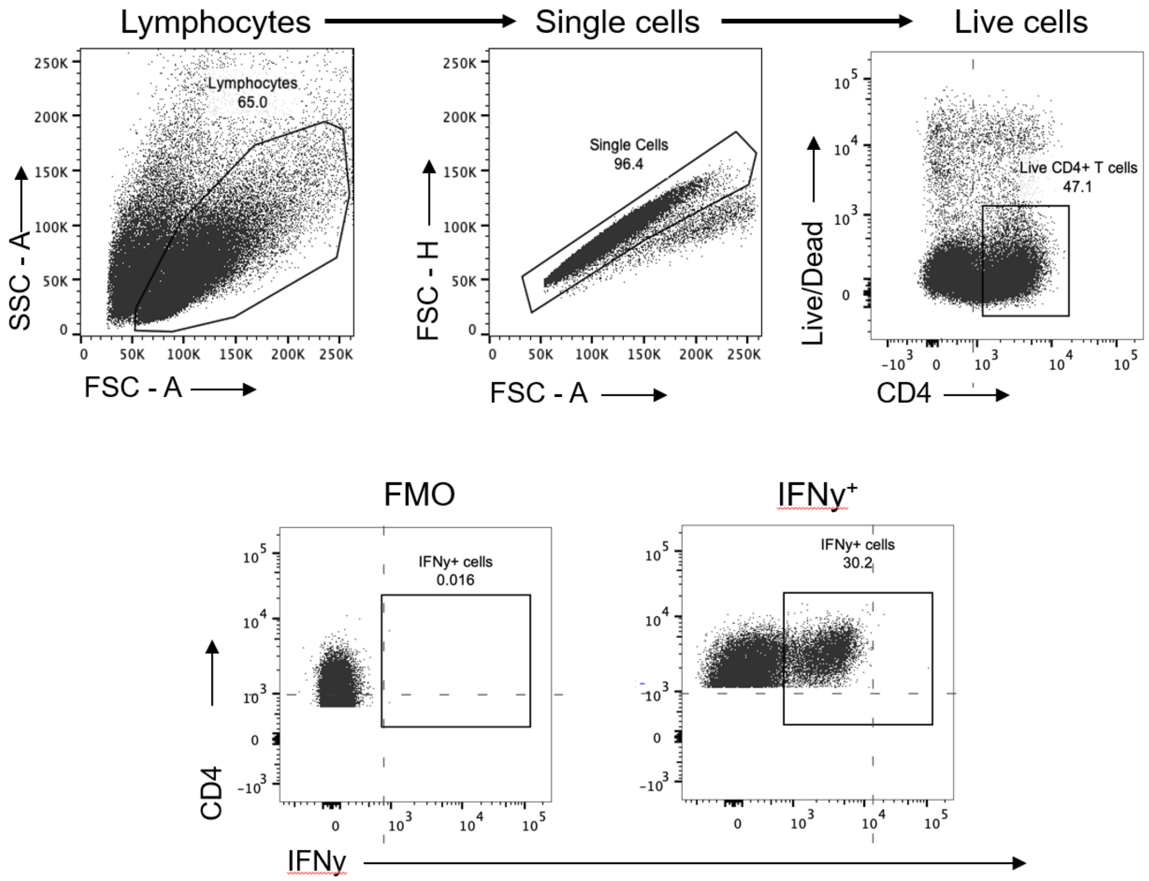

**Supplementary Table 1. Cohort Characteristics**		
	**Advanced NSCLC Cohort (n=46)**	**Control (n=8)**
Age (years)	72.3 +/- 1.8	70.7 +/- 0.8
Gender (% male)	73%	60%
**Advanced NSCLC Cohort (n=46)**		
Mutation Status*		
PD-L1 < 50%	56.5% (n=26)	
PD-L1 > 50%	43.5% (n=20)	
Treatment Arm		
Anti-PD1 (Pembrolizumab or Nivolumab)	32.6% (n=15) [12 Pembrolizumab, 3 Nivolumab]	
Anti-PD1 + Chemotherapy	52.2% (n=24)	
Anti-PDL-1 (Atezolizumab)	6.5% (n=3)	
Anti-PDL-1 + Avastin + Chemotherapy	8.7% (n=4)	
Tumour Stage		
IASLC Stage IIIb	8.7% (n=4)	
IASLC Stage IIIc	26.1% (n=12)	
IASLC Stage IVa	41.3% (n=19)	
IASLC Stage IVb	23.9% (n=11)	
Histology		
Adenocarcinoma	67.4% (n=31)	
Squamous Cell Carcinoma	32.6% (n=15)	
Immune-Related Adverse Events**		
Grade 3-4	17.4% (n=8)	
Target Organ		
Colon	6.5% (n=3)	
Lungs	2.2% (n=1)	
Kidneys	2.2% (n=1)	
Other***	6.5% (n=3)	
Number of Organs affected		
0	82.6% (n=38)	
1	15.2% (n=7)	
2	2.2% (n=1)	

*All patients were eGFR, ALK, ROS, BRAF wildtype except those who underwent QUAD therapy treatment with Atezolizumab, Avastin and Chemotherapy following TKI failure (eGFR+ve)

**No significant differences in any of the cohort characteristics between toxicity and non-toxicity patients

***Other includes arthritis, arthralgias, polymyalgia rheumatica and dry eyes

Supplementary Table 2. Mass Cytometry Antibody Panels

Base B cell Panel

Metal Tag	Antibody	Clone	Source	Dilution (µL)
89Y	CD45	HI30	Fluidigm	0.6
141Pr	CD3	UCHT1	Fluidigm	0.75
142Nd	CD19	HIB19	Fluidigm	0.4
144Nd	CD38	HIT2	Fluidigm	0.5
145Nd	CD81	5A6	Fluidigm	0.5
146Nd	IgD	IA6-2	Fluidigm	0.5
147Sm	CD20	2H7	Fluidigm	0.6
148Nd	CD8a	SK1 (Custom)	Biolegend	0.75
149Sm	CD25 (IL2R)	2A3	Fluidigm	0.7
150Nd	CD138	DL-101	Fluidigm	0.75
151Eu	HLA-DR	G46-6	Fluidigm	0.25
152Sm	CD21	BL13	Fluidigm	0.5
153Eu	Ig Lambda	MHL-38 (Custom)	Biolegend	0.5
154Sm	IgG R10	R10 (Custom)	Biolegend	0.5
155Gd	CD279 (PD-1)	EH12.2H7	Fluidigm	0.75
156Gd	CD274 (PD-L1)	29E.2A3	Fluidigm	0.75
158Gd	CD10	HI10a	Fluidigm	0.7
159Tb	CD22	HIB22	Fluidigm	0.5
160Gd	Ig Kappa	MHK-49	Fluidigm	0.5
161Dy	CD5	UCHT2 (Custom)	Biolegend	0.5
162Dy	CD79B	CB3-1	Fluidigm	0.5
163Dy	BCL-6	K112-91	Fluidigm	0.5
164Dy	CD95/Fas	DX2	Fluidigm	0.5
165Ho	CD40	5C3	Fluidigm	0.5
166Er	IL-10	JES3-9D7	Fluidigm	1
167Er	CD27	L128	Fluidigm	0.4
168Er	Ki-67	B56	Fluidigm	0.6
169Tm	CD24	ML5	Fluidigm	0.5
170Er	TGF-Beta	TW4-6H10 (Custom)	Biolegend	1
171Yb	CD185 (CXCR5)	51505	Fluidigm	0.75
172Yb	IgM	MHM-88	Fluidigm	0.3
175Lu	CD28	CD28.2 (Custom)	Biolegend	0.5
176Yb	CD4	RPA-T4	Fluidigm	0.5
209Bi	CD16	3G8	Fluidigm	0.25

Breg/Tfh Panel

Metal Tag	Antibody	Clone	Source	Dilution (µL)
89Y	CD45	HI30	Fluidigm	0.6
141Pr	CD3	UCHT1	Fluidigm	0.75
142Nd	CD19	HIB19	Fluidigm	0.4
143Nd	ICOS (CD278)	C398.4A	Fluidigm	0.5
144Nd	CD38	HIT2	Fluidigm	0.5
145Nd	CTLA-4 (CD152)	14D3 (Custom)	Biolegend	1
146Nd	IgD	IA6-2	Fluidigm	0.5
147Sm	IL-21	3A3-N2 (Custom)	Biolegend	0.6
148Nd	CD8a	SK1 (Custom)	Biolegend	0.75
149Sm	CD25 (IL2R)	2A3	Fluidigm	0.7
150Nd	CD71	OKT-9 (Custom)	Biolegend	0.5
151Eu	CD40L	24-31	Fluidigm	0.75
152Sm	CD1d	51.1 (Custom)	Biolegend	0.75
153Eu	TIGIT	MBSA43	Fluidigm	0.75
154Sm	IL-4	MP4-25D2 (Custom)	Biolegend	0.6
155Gd	CD279 (PD-1)	EH12.2H7	Fluidigm	0.75
156Gd	CD274 (PD-L1)	29E.2A3	Fluidigm	0.75
158Gd	CXCL13	53610 (Custom)	Bio-Techne	1
159Tb	IL-35	27537 (Custom)	Bio-Techne	0.6
160Gd	CD39	A1	Fluidigm	0.75
161Dy	CD5	UCHT2 (Custom)	Biolegend	0.5
162Dy	TIM-1	1D12 (Custom)	Biolegend	0.75
163Dy	BCL-6	K112-91	Fluidigm	0.5
164Dy	CD95/Fas	DX2	Fluidigm	0.5
165Ho	CD40	5C3	Fluidigm	0.5
166Er	IL-10	JES3-9D7	Fluidigm	1
167Er	CD27	L128	Fluidigm	0.3
168Er	Ki-67	B56	Fluidigm	0.6
169Tm	CD24	ML5	Fluidigm	0.5
170Er	TGF-Beta	TW4-6H10 (Custom)	Biolegend	1
171Yb	CD185 (CXCR5)	51505	Fluidigm	0.75
172Yb	IgM	MHM-88	Fluidigm	0.3
173Yb	CD73	AD2 (Custom)	Biolegend	0.5
174Yb	HLA-DR	L243	Fluidigm	0.3
175Lu	CD28	CD28.2 (Custom)	Biolegend	0.5
176Yb	CD4	RPA-T4	Fluidigm	0.5
209Bi	CD16	3G8	Fluidigm	0.25

Supplementary Table 3. Antibodies and their corresponding fluorophores used for intracellular flow cytometry

Marker	Biolegend Fluorophore
Fixability Viability Dye eFluor 780	Equivalent to APC-eFluor 780 or APC Alexa Fluor 750
IL-2	Ax647
IL-6	PE/Dazzle 594
IL-10	Ax488
IL-17	PE
TNF-α	BV421
IFN-γ	PE-Cy7
Cell Trace Violet	401-450 Excitation
