# Supplementary material for: Regulatory B cell repertoire defects predispose lung cancer patients to immune-related toxicity following checkpoint blockade
Source: Nat Commun. 2022 Jun 7;13:3148. doi: 10.1038/s41467-022-30863-x (PMC9174492; doi:10.1038/s41467-022-30863-x)
Supplement: Supplementary file 2 — Reporting Summary [file 41467_2022_30863_MOESM2_ESM.pdf]

nature portfolio

Corresponding author(s): Gary Middleton

Last updated by author(s): Mar 30, 2022

Reporting Summary

Nature Portfolio wishes to improve the reproducibility of the work that we publish. This form provides structure for consistency and transparency in reporting. For further information on Nature Portfolio policies, see our Editorial Policies and the Editorial Policy Checklist.

Statistics

For all statistical analyses, confirm that the following items are present in the figure legend, table legend, main text, or Methods section.

n/a	Confirmed	
☐	☒	The exact sample size (*n*) for each experimental group/condition, given as a discrete number and unit of measurement
☐	☒	A statement on whether measurements were taken from distinct samples or whether the same sample was measured repeatedly
☐	☒	The statistical test(s) used AND whether they are one- or two-sided<br>*Only common tests should be described solely by name; describe more complex techniques in the Methods section.*
☐	☒	A description of all covariates tested
☐	☒	A description of any assumptions or corrections, such as tests of normality and adjustment for multiple comparisons
☐	☒	A full description of the statistical parameters including central tendency (e.g. means) or other basic estimates (e.g. regression coefficient) AND variation (e.g. standard deviation) or associated estimates of uncertainty (e.g. confidence intervals)
☐	☒	For null hypothesis testing, the test statistic (e.g. *F*, *t*, *r*) with confidence intervals, effect sizes, degrees of freedom and *P* value noted<br>*Give P values as exact values whenever suitable.*
☒	☐	For Bayesian analysis, information on the choice of priors and Markov chain Monte Carlo settings
☐	☒	For hierarchical and complex designs, identification of the appropriate level for tests and full reporting of outcomes
☒	☐	Estimates of effect sizes (e.g. Cohen's *d*, Pearson's *r*), indicating how they were calculated

Our web collection on statistics for biologists contains articles on many of the points above.

Software and code

Policy information about availability of computer code

Data collection	All data collection was done prospectively as part of observational study. Data was analysed using R, version 4.0.3.
Data analysis	R Studio (v4.0.3), R 4.0.3 was used for all data analysis, code pipelines were adapted from CATALYST, diffCYT, FlowSOM, UMAP packages and Nowicka et al pipeline as described in the literature and detailed in the methodology. Mass and flow cytometric analysis was carried out using MRC Cytobank software (Tree Star Inc.). Boxplot visualisation was carried out using the ggplot2 visualisation engine through the ggpubr (v0.4.0) package. The authors declare that the code for reproducibility of data are publicly available. Although the code was adapted from various sources, the underlying code itself was not modified or changed in any way and is readily available from the sources cited. The code can be made available from the corresponding author upon reasonable request.

For manuscripts utilizing custom algorithms or software that are central to the research but not yet described in published literature, software must be made available to editors and reviewers. We strongly encourage code deposition in a community repository (e.g. GitHub). See the Nature Portfolio guidelines for submitting code & software for further information.

Data

Policy information about availability of data

All manuscripts must include a data availability statement. This statement should provide the following information, where applicable:

- Accession codes, unique identifiers, or web links for publicly available datasets
- A description of any restrictions on data availability
- For clinical datasets or third party data, please ensure that the statement adheres to our policy

Mass and flow cytometry data: the data that support the findings of this study are available from the corresponding author upon reasonable request. This is largely

owing to file size and logistics of patient confidentiality, reverse pseudonymisation and need for data to be kept at specific academic/research sites in line with the policies from individual trial protocols. Source data are provided with this paper.

Field-specific reporting

Please select the one below that is the best fit for your research. If you are not sure, read the appropriate sections before making your selection.

☒ Life sciences ☐ Behavioural & social sciences ☐ Ecological, evolutionary & environmental sciences

For a reference copy of the document with all sections, see nature.com/documents/nr-reporting-summary-flat.pdf

Life sciences study design

All studies must disclose on these points even when the disclosure is negative.

Sample size	This was an exploratory analysis therefore sample sizes were not taken, as it would have been difficult to estimate the power of the study. However the following considerations have been taken into account. In 1995 Browne recommended a sample of 30 subjects or more as a general rule for pilot studies when the aim is to obtain variance estimates for a sample size calculation sample size for a bigger Randomised Controlled Trial. Kieser and Wassmer investigated the theoretical basis for Browne's recommendation and concluded that a sample size of 40 will be adequate for applying Browne's method in the subsequent sample size calculation. We therefore chose an intended sample size of at least 40 patients for a given disease area. This sample size will allow the mean value for a standardized, normally distributed primary outcome measure to be estimated to within ±4% with 90% confidence, i.e. 90% confidence interval (CI) width of 8%, assuming a relative standard deviation of 10%. Recruitment of at least 44 patients total will allow for the sample size of 40 to be met whilst allowing for 10% dropout.
Data exclusions	Exclusions included those patients who had been treated previously with any form of immunotherapy, previous cancers or unwillingness to participate in the study.
Replication	Findings were replicated in a separate completely independent cohort from a separate institute using the same CyTOF protocol. Technical replicates for each participant were performed at least twice independently and were successful in all cases.
Randomization	This was an observational translational study with prospective participant recruitment, not a randomised clinical trial. Following pseudonymisation of all samples, when selecting cases for analysis, all were selected in a randomised manner with clinical metadata not known to investigators, this allowed for controlling of covariate clinical factors.
Blinding	Investigators were blinded to group allocation and patient outcome during data analysis through pseudonymisation of all patient samples.

Reporting for specific materials, systems and methods

We require information from authors about some types of materials, experimental systems and methods used in many studies. Here, indicate whether each material, system or method listed is relevant to your study. If you are not sure if a list item applies to your research, read the appropriate section before selecting a response.

Materials & experimental systems

n/a	Involved in the study	
☐	☒	Antibodies
☒	☐	Eukaryotic cell lines
☒	☐	Palaeontology and archaeology
☒	☐	Animals and other organisms
☐	☒	Human research participants
☒	☐	Clinical data
☒	☐	Dual use research of concern

Methods

n/a	Involved in the study	
☒	☐	ChIP-seq
☐	☒	Flow cytometry
☒	☐	MRI-based neuroimaging

Antibodies

Antibodies used	All antibodies used including clone, metal tag, source and individual dilution are specified in Supplementary Table 2.
Validation	All antibodies were biologically validated by the manufacturer, internal experimental validation was performed on healthy control subjects to determine appropriate titrations for the antibody cocktail. Those antibodies that were readily bought from Fluidigm Inc. had undergone specific biological validation such that each reagent is detected and quantified with cytometry by time-of-flight mass spectrometry in the CyTOF system. The high purity and choice of metal isotopes ensure minimal background noise from signal overlap or endogenous cellular components. Those antibodies that were custom conjugated, technical advice was sought from the fluidigm field application specialists and conjugation was carried out using the Fluidigm metal-labelling kits. Individual titrations were carried out on healthy controls and run as per the fluidigm validation protocol on the CyTOF system as detailed above.

Human research participants

Policy information about studies involving human research participants

Population characteristics	These are detailed in Supplementary Table 1. Patient Characteristics.
Recruitment	Peripheral blood mononuclear cells (PBMC) from age-matched healthy donors were obtained from the Clinical Immunology Service at the University of Birmingham (UoB). Primary blood samples from advanced Non-Small Cell Lung Cancer (NSCLC) patients were obtained before and after the first cycle of treatment with checkpoint blockade immunotherapy either as monotherapy or in combination with chemotherapy. Written consent was provided under the UoB Research Ethics Approval, protocol 10/H0501/39. Tumour stage and histological subtype with molecular profiling was determined by a radiologist and pathologist respectively (S1). The PAIR study was used to provide the validation arm samples, this was based at Guys and St Thomas' NHS foundation trust. All uses of human material from this study was approved by the Guy's and St. Thomas' Research Ethics approval (REC reference 17/LO/1950) and written consent was provided by all participants recruited under this research approval. Specific patient recruitment was carried out according to the inclusion criteria, i.e. those who were able to give informed consent and have advanced stage lung cancer amenable to treatment with immunotherapy. Exclusion criteria were the same as those who were not able to undergo the treatment. Patients were identified from the waiting lists and screened for eligibility by the research fellow at respective sites. The recruitment technique employed was purposive sampling selection thus in keeping with our methodology and theoretical framework.
Ethics oversight	The ethical boards that approved the research studies at each site were the NRES Committee South Central (Hampshire B) (REC 10/H0501/39) and the London - Camberwell St Giles Research Ethics Committee (REC reference 17/LO/1950).

Note that full information on the approval of the study protocol must also be provided in the manuscript.

Flow Cytometry

Plots

Confirm that:

- ☒ The axis labels state the marker and fluorochrome used (e.g. CD4-FITC).
- ☒ The axis scales are clearly visible. Include numbers along axes only for bottom left plot of group (a 'group' is an analysis of identical markers).
- ☒ All plots are contour plots with outliers or pseudocolor plots.
- ☒ A numerical value for number of cells or percentage (with statistics) is provided.

Methodology

Sample preparation	Antibodies used, and their corresponding fluorophores, are shown in Figure S3. For cytokine detection, cells were re-stimulated in the final three (CD19+ only) or five (co-culture) hours of culture with PMA (1mg/mL) and Ionomycin (1mg/mL, Sigma) in the presence of Brefeldin A, and GolgiStop (both 1mg/ml, both BD Biosciences). Cells were harvested and incubated with Live/Dead, then washed and fixed in 4% paraformaldehyde (Fixation Buffer, Biolegend) for 15 minutes at room temperature. Cells were then incubated with the appropriate volume of antibody, diluted in 0.1% Saponin in 0.5% BSA in PBS for 45 minutes at room temperature. Cells were acquired using a BD FACSCanto II (BD Biosciences), and analysis was conducted using MRC Cytobank software (Tree Star Inc.). Gating was carried out using either a fluorescence minus one control, and/or unstimulated cells.
Instrument	HeliosTM Mass Cytometer (Fluidigm Inc.)
Software	As above, higher downstream analysis performed in R Studio, R v4.0.2 using ggpubr packages for data visualisation.
Cell population abundance	PBMCs were isolated as described. CD4+ T cells and CD19+ B cells were subsequently isolated by magnetic cell sorting, based on CD4 microbead positive selection and subsequent CD19 negative selection (MACS, Miltenyi Biotech); purity was consistently >98%.
Gating strategy	Use MRC Cytobank to gate<br>1. Gate Singlets (FSC-H [y axis], FSC-A [x axis])<br>2. Gate Lymphocytes (SSC-A [y axis], FSC-A [x axis])<br>3. Gate Live cells (L/D channel [y axis], FSC-A [x axis])<br>A gating strategy figure can be included if required as supplementary.

☒ Tick this box to confirm that a figure exemplifying the gating strategy is provided in the Supplementary Information.
